# Opto-Mechanical Eye Models, a Review on Human Vision Applications and Perspectives for Use in Industry

**DOI:** 10.3390/s22197686

**Published:** 2022-10-10

**Authors:** André Rino Amorim, Boris Bret, José M. González-Méijome

**Affiliations:** 1 Clinical and Experimental Optometry Research Lab, Center of Physics, University of Minho, 4710-057 Braga, Portugal; 2 Bosch Car Multimedia Portugal, S.A., 4705-285 Braga, Portugal

**Keywords:** optomechanical artificial eye model, in vitro eye measurements, imaging, image quality, human eye

## Abstract

The purpose of this review is to aggregate technical information on existent optomechanical eye models (OME) described in the literature, for image quality assessment in different applications. Several physical eye models have been reviewed from peer-reviewed papers and patent applications. A typical eye model includes an artificial cornea, an intraocular lens or other lens to simulate the crystalline lens, an aperture as the pupil, and a posterior retinal surface, which may be connected to a light sensor. The interior of the eye model may be filled with a fluid to better emulate physiological conditions. The main focus of this review is the materials and physical characteristics used and the dimensional aspects of the main components including lenses, apertures, chambers, imaging sensors and filling medium. Various devices are described with their applications and technical details, which are systematically tabulated highlighting their main characteristics and applications. The models presented are detailed and discussed individually, and the features of different models are compared when applicable, highlighting strengths and limitations. In the end there is a brief discussion about the potential use of artificial eye models for industrial applications.

## 1. Introduction

The human eye is an organ that combines singular anatomical and histological characteristics with complex physiological properties to develop its function as an image formation “device”. Among the multiple conditions that can impair vision, the cataract is the most common one, and is the most frequent surgical intervention. The development of artificial intraocular lenses (IOLs) to compensate the removal of the natural crystalline lens after cataract requires a detailed study of the optical properties of the human eye. Also common are refraction and aberration conditions, such as myopia or astigmatism, and optical benches to design and test spectacles or contact lenses (CLs) are also widely required. In addition, with the advance of optical testing devices and techniques for physiological applications, there has also been an increased interest in providing dummy eyes to validate CLs and IOLs. New treatments such as laser interventions have also been tested in vitro.

Compared to other physiological systems, there is a strong interest in physical modeling of the optics of the eye. Due to their small dimensionality and complexity, mere computer theoretical models are usually not practical for assessing quality of vision, designing optical implants or measurement devices, or planning and monitoring treatments. One of the main challenges is to be able to predict the subjective response of a patient prior to actually altering his/her vision, either surgically or by external means such as spectacles or contact lenses. An additional problem is the fact that the eye characteristics present large inter-individual variability, which means that the models need to be highly customizable. Thus, the ability to accurately predict the in vivo performance of constantly changing ocular parameters has many obvious advantages over theoretical computer models, with the goal of reducing the time, cost, and uncertainty involved in efforts to improve visual quality. For these reasons, there has been an increase in the development of physical models of the eye in the last decades, in order to conduct preliminary tests (bench testing) of image formation capabilities of optical devices for physiological applications. This interest was boosted by the increased complexity of the design in recent years.

An optomechanical eye model (OME), sometimes called optomechanical artificial eye model (OMAE), or physical artificial eye model, is a customizable system that tries to mimic the anatomy and function of the human eye in vitro. Various efforts had been made to build realistic physical eye models. While initial devices were based mainly on the well-known Gullstrand reduced eye model [1], nowadays, eye models are way more complex, detailed and varied. Eye models are widely used as benchmarks to evaluate the performance of spectacles, contact lens (CLs) and intraocular lenses (IOLs), and are also used to test and calibrate optical measurement devices, new surgical interventions and for use in research studies.

Besides the fields of physiological optics, optometry, ophthalmology or biomedical engineering, the global mass production industry has taken advantage of the advances in artificial vision capability to assist in quality control, as well as other parts of the production, storage and delivery processes. This is usually conducted with devices developed for other purposes, which might provide high imaging performance capabilities, but are not intended to reproduce the visual capabilities of the human eye. Additionally, there are tasks that still require the intervention of a human observer to analyze if the products carry defects that could be detected by a human observer. Using the same automatic processes comes with the risk of over-detecting failures that would be never detected by a human observer. Therefore, there is a growing interest to use eye models that can mimic the imaging characteristics of the human eye to replace human observers in tedious processes that require human intervention.

The present review summarizes some of the physical artificial eye models developed in the field of physiological optics to test and mimic the human vision conditions and the effect of artificial devices to compensate vision problems or to test the effects of surgical interventions. Considering that the main applications are in the domain of physiological optics and biomedical engineering connected with applications in the field of visual optics, optometry and ophthalmology, a literature review was conducted using the “PubMed” database as the main source, complemented by searches in different patent databases. Only publications in the English language were considered, despite eye models in other languages having also been found [2]. An additional search was performed for components that may be useful for use in eye models, mainly tunable lenses to mimic the crystalline lens activity or curved sensors to emulate the retinal shape. Although only in vitro physical models are considered, some schematic theoretical models will also be mentioned, when necessary, since they are important references for physical models. Among the artificial eye models reviewed, the main focus is on the subset of devices for human vision applications, while briefly mentioning devices for other applications.

A total of 70 articles and eight patents were found, and are listed in Table 1. If the same eye model is presented in more than one publication involving approximately the same research team, the most relevant one will be preferred, while still mentioning the related publications. After excluding those that were not related to applications in human vision, a total of 13 articles and four patents were selected for a deeper analysis. Nonetheless, the remaining publications will also be considered, but in less detail.

Before discussing the different eye models, the different model types and their main applications are summarized in the next section. Later, a general overview of the basic components included in an artificial model is presented, followed by a debate on how they are assembled. A discussion section includes a general overview of the performance, advantages and disadvantages of the different devices presented, as well as some guidelines, suggestions, and personal comments about physical eye models.

## 2. Physical Eye Models and Their Applications

Based on theoretical simulations, Gliddon (1929) [3] showed that it was possible to build an optical replica of the human eye for the study of the retinal image. The replica consisted of a copper cell which could be filled with fluid, included a movable mount with a glass retina, replaceable diaphragms to mimic the iris and pupil, double-shell glass lenses with water in between, to model the cornea and crystalline lens, that were placed in magnetic holders that could be moved so that the cornea or lens, or both, could be tipped and decentered. The model was tested on a bench, and the image at the retinal glass was examined with the help of an objective and an eyepiece. This eye model was very detailed for the time it was constructed, and included many of the foundations of modern eye models.

Other eye models followed. In 1978, Arell and Kolari [4] constructed a low-cost adjustable artificial eye model with approximately the real focal length, to use as an object in direct and indirect ophthalmoscopy, as a laboratory experiment for medical and biology students.

In 1987, Heath and collaborators [5] wanted to map distortions associated with ophthalmic lenses. They created a device that consisted of a pinhole camera and a lens holder, where an ophthalmic lens could be placed. The lens mount was designed to include various pantoscopic tilt positions and also allowed for horizontal and vertical rotation around a point to simulate the lens/eye relation when the eye position is changed. The pinhole camera represented the human eye, while its thin film could be viewed as the retina. Despite not being sophisticated and requiring prolonged light exposure, this simple device was one of the first eye model precursors to make it possible to capture and record a retinal image.

In 1992, Rudnicka et al. [6] constructed a physical eye model based on the Bennett and Rabbetts schematic eye [81]. It incorporated a cornea, a lens, and a spherical fundus. The vitreous chamber depth could be precisely varied to produce axial ametropia from −11D to −17D [6]. The optical performance of the model eye was tested with a refractometer, and then the device was used to investigate the relationship between the actual size of a fundus feature and its photographic image in two different fundus cameras.

Still in 1992, Portney [82] presented a method for in vitro IOL inspection, by showing theoretically that a wet eye cell can be designed to be optically equivalent to theoretical eye models. The cell model used was a combination of a wet-cell with a conjugation lens to act as the cornea. A wet-cell is typically a transparent glass cuvette filled with water, where an IOL is immersed for analysis. The use of water cells to evaluate IOLs had been previously described by Holladay [8,9], and is currently a widely popular technique for this application, existing several in vitro eye models based on them.

Today, the International Organization for Standardization (ISO) 11979-2 [83] for evaluation of imaging quality of IOLs provides a model eye with an essentially aberration-free artificial cornea. It consists of a wet-cell with two parallel glass plates made of BK7 glass, which are filled with saline solution with refraction index of 1.336 (546.1 nm) at 35 °C (optional heating) [84]. Several artificial eye models are based on this standard, and some will be mentioned later in this review. A more recent version of the same ISO standard introduced an alternative cornea with spherical aberration, to better mimic the human eye [85].

Artificial eye models have been used for various types of applications. The most common is to evaluate lenses, mainly IOLs, but also CLs or spectacles. Eye models are also widely used for the validation and calibration of ophthalmic equipment. Another type of applications is the evaluation of measurement methods applied to ophthalmology, and the in vitro testing of new surgical treatments.

Another great interest of using physical eye models is to simulate/model selected optical characteristics of the natural eye, for research applications. For example, there have been reported applications to simulate eye measurements in a controlled in vitro environment, to model function such as accommodation, and to simulate human vision under various optical conditions, such as when driving at night, reading text, or recognizing objects. Finally, eye models have also been used for teaching and education purposes, and for potential use in optical design. Table 2 summarizes the main applications of each device presented in this review. This does not mean that the devices are not fit for other uses, but only that these are their intended applications as reported in the publications reviewed.

Eye models for non-optical applications, such as dummy eye models for surgical training, to evaluate eye tracking devices, to validate ultrasound measurements, or to simulate eye movements, will not be considered in this review (see, for example, refs. [86,87,88,89,90]).

## 3. Measurement Modalities of Eye Models

Considering only the physical eye models for optical-related applications, they can be divided into six main measurement modalities: wavefront measurements; single-pass measurements; double-pass measurements; Scheimpflug or Purkinje imaging; fundus imaging and retinoscopy; and biometry and optical coherence tomography (OCT) measurements. Other non-optical modalities, such as ultrasound measurements (Ref. [52] reported ultrasound biomicroscopy for device characterization, and a dummy eye model for ultrasound biometry is commercially available at [90]), thermal measurements [70], or mechanical and structural characterizations [55], will not be considered in this review, unless the model itself presents relevant features related to optical measurements. Table 3 organizes the main modalities of the respective devices mentioned in this review. Some devices are “multi-modal”, and more than one measurement modality was reported in the respective publications [28,35,37,41,42,48,52,55,64,70,72,80].

This review is focused on the devices used for single-pass complex scene imaging, which are the ones most associated with human vision applications. The next sub-sections briefly discuss the optical modalities and respective eye models existent in literature. The following section focuses on human vision.

### 3.1. Double-Pass Measurements

The double-pass (DP) technique is a half-century-old method based in the recording of the retinal image after passing twice through the ocular media, i.e., before and after retinal reflection. This method provides accurate estimates of the eye’s image quality, in the form of metrics such as the modulation transfer function (MTF) or the optical transfer function (OTF), which gives information on the overall optical performance of the eye, including all the optical defects involved in retinal image degradation, such as diffraction, aberrations, and scattering [91]. This method is particularly powerful in evaluating many of the conditions that especially affect scattering, but is now less popular for wavefront measurements.

While most eye measurements involve the light to pass twice through the eye, in a double-pass experiment, both passes provide information about the eye optics. Measurements such as fundus imaging only use information from the second pass, and some wavefront measurements are double-pass, while others are second-pass (such as HS aberrometry). This section includes only methods that rely on both passes and do not involve wavefront measurements (discussed in the next sub-section), focusing instead on measures such as the MTF. In addition, only when light is reflected by the retina surface is it considered a double-pass. Scheimpflug and Purkinje reflections are therefore not considered as such.

Eye models for double-pass measurements are characterized by the use of a camera sensor which is located usually outside and in front of the eye (mirrors are used when that is not possible), in order to capture a retinal image that was reflected back to outside the eye. As in wavefront measurements, the retina is also commonly a diffuse reflector. The model previously described by Rudnicka et al. [6] was characterized using autorefractometry, despite this was not its main purpose. The eye model described by Pujol et al. [8] consisted of a simple setup using an achromatic lens as the cornea and a diffuser which could rotate in order to avoid the influence of speckle. Additional lenses with no spherical power and different cylindrical powers were applied to evaluate astigmatism based on cross-cylinder examination methods. The model of García-Guerra [59] previously mentioned double-pass techniques that are also used. The patent by Niven [79] described an eye model for development purposes, with an orange acrylic endcap for use with double-pass measurements. Another simple model was built by Goncharov et al. [35] to measure wavefront aberrations through double-pass experiments using an interferometer, whose data would be used to fit theoretical eye models, in an inverse optical design approach.

### 3.2. Wavefront Measurements

Wavefront measurements have gained increasing popularity in the last two decades for measuring ocular optical aberrations, as they can objectively characterize the properties of optical systems, including the human eye that is physiological aberrated by the shape of the refractive surfaces. Such aberrations affect the image quality and the visual performance. Additionally, several corrective and surgical interventions further modify the aberration wavefront properties of the eye. Probably the most well-known wavefront sensor is the Hartmann-Shack (HS) sensor, present in most clinical aberrometers. The main drawback of wavefront sensors is the lack of information on scattering [91].

Being an increasingly popular technique, it is expected that there are quite a few artificial eye models related with wavefront measurements. The main characteristic of these models is the retinal surface, which is usually a diffuser that can often rotate in order to avoid speckle noise and destroy the spatial coherence of input light. The light source is typically a laser, while the measurement equipment is typically the Hartmann-Shack (HS) sensor. The analysis often consists in the wavefront reconstruction quantitative characterization of aberrations, and evaluation of metrics such as Zernike or Seidel coefficients.

Eye models have been used together with wavefront measurements in order to validate wavefront measuring devices [15,78], for adaptative optics testing using HS sensors [24], or to evaluate ophthalmic lens [32,41,44,77]. These models are usually designed in such a way it is easy to add and control aberrations. For example, Yamaguchi et al. [78] used a phase plate for that purpose, while [24] used a tunable crystalline lens, and Letfullin et al. [17] and Galetskiĭ et al. [21] used biomorph mirrors to reproduce aberrations and their temporal dynamics in real time. More minimalistic models have also been used, such as by Shen et al. [34] or Guerra et al. [59]. The latter used a combination of data obtained from double-pass and HS measurements to perform estimations of aberrations and scattering in the human eye. The complete model of Bakaraju et al. [37] also allowed a mode for wavefront measurements using a COAS aberrometer, and will be described in a later section. Norrby et al. [23] also presented wavefront measurements using his device. Esteve-Taboada et al. [57] and Drauschke et al. [48] also used wavefront measurements, but only to characterize the eye model aberrations. Moreno-Barriuso et al. [9] used a set of models where HS wavefront measurements were compared with laser ray-tracing (LRT) methods, in both a single-pass and a double-pass manner.

**Figure 1:**
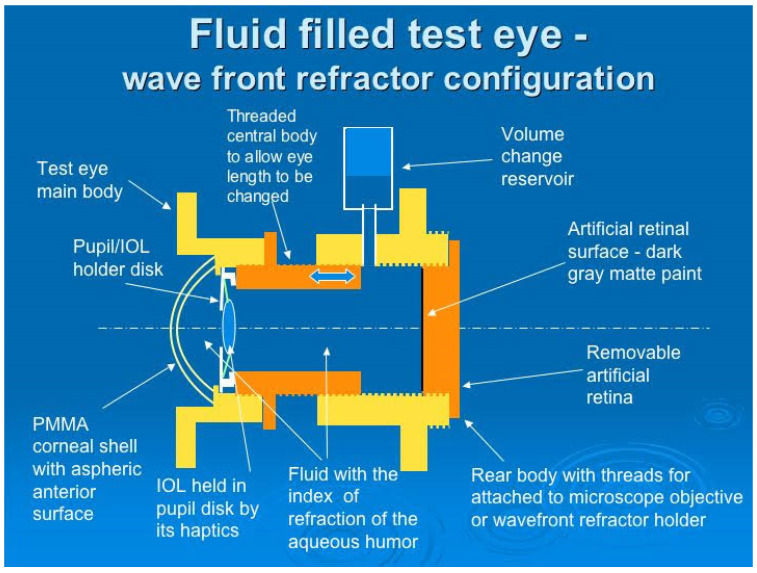
A cross-sectional view of the eye model used by Campbell. The IOL being tested is shown suspended from its haptics in the pupil disk just as it is in a human eye. Journal of Refractive Surgery 2008;24(3):308–311 ([28]). Originally published by and reprinted with permission from SLACK Incorporated.

A model that is more detailed was built by Campbell [28]. It was designed to investigate whether wavefront measurements obtained in eyes that have implanted multifocal IOLs gave reliable guidance on creating laser refractive surgical treatments to remove residual refractive error from those eyes. This system allowed for both HS or single-pass measurements, by exchanging a painted surface retina by a glass window connected to a charge-coupled device (CCD) camera through a microscope. However, the latter was used only to set the system for emmetropia with the help of a Badal optometer, by adjusting the length of the eye until the best point spread was observed with the camera system. Then, the transparent window was replaced by the solid retina for the aberration measurements. A schematic representation of this device is in Figure 1.

**Figure 2:**
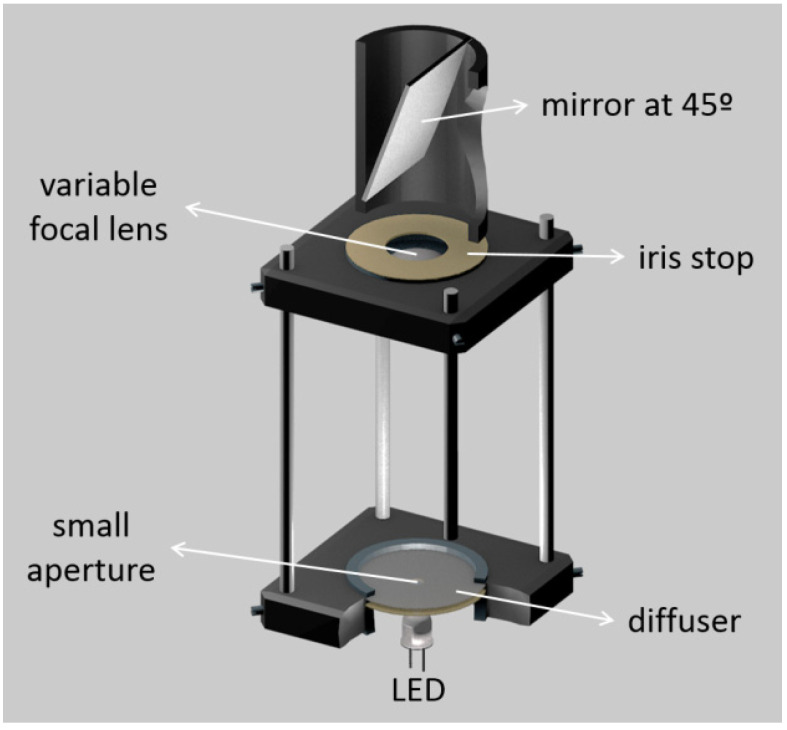
Three-dimensional rendering of the device used by Esteve-Taboada et al. For a description of its components, see text. Optics Express 2015, 23, 19396 ([57]). Originally published by and reprinted with permission from Optica Publishing Group.

Wavefront measurements were also performed in reverse direction, using a light source at the level of the retina and the sensor in front of the eye. These measurements are typically called second-pass measurements. Esteve-Taboada et al. [57] designed an opto-mechanical artificial eye for second-pass aberrometer measurements, with the purpose of avoiding the disadvantages of double-pass measurements. A near infrared (NIR) light emitting diode (LED) was used together with a diffuser, to function as an active retina. A diaphragm centered with respect to the rest of the device’s optical components made it possible to limit the amount of light emitted by the retina. The device also had accommodative ability through the use of a lens with an electronically variable focal length, to control the refractive state of the device. A second diaphragm iris (artificial pupil) was added just after the variable lens to acts as the aperture diaphragm of the system (Figure 2). If desired, a wet-cell with an IOL could be placed inside, near the artificial pupil. A commercial HS aberrometer was used to characterize the aberrations of the system. 

Coughlan et al. [64] presented an opto-mechanical artificial eye that could be used for examining multi-wavelength ophthalmic instruments. The model was based on the schematic eye of Escudero-Sanz and Navarro [92]. The retina was modeled with a semi-transparent disc and field-stop, and its design offered two modes of operation: HS aberrometry to measure longitudinal chromatic aberration and the possibility of inducing refractive error, for on-axis testing; and second-pass measurements, by back-illuminating the retinal surface and using a CMOS detector, to investigate off-axis chromatic aberration. Due to the ability of performing second-pass measurements on the eye model, the authors said it had a “dispersive design”. The invention described by Altmann et al. [77] also mentions back-illumination.

### 3.3. Single-Pass Measurements

Single-pass measurements would be the most desired modality for most applications, as they are simpler and have less limitations compared to double-pass measurements, because there is not the problem of specular reflections occurring in the different refractive surfaces. Unfortunately, these measurements are not realistic, as they require measuring light at the retinal surface, which is not possible in vivo with human eyes. The only single-pass measurement we can do in vivo today is still our own natural retinal processing. Therefore, eye models offer a great tool for this purpose, as we can freely implement a CCD camera or other sensor in the artificial eye. For this reason, it is not surprising that eye models aimed at single-pass models are the ones most commonly reported in literature (Table 2).

The most common performance metric to be measured using the single-pass technique is the modulation transfer function (MTF), which is determined by using the magnitude of the Fourier domain analysis. It reveals how different spatial frequencies are handled by the optical system, and can be measured directly via a cross-target or point-/spot-target to obtain the line or the point spread function (LSF, PSF) resulting from imaging a thin line or point of light, respectively [84]. This can be done using trans-illuminated frequency charts (MTF tests), such as the ones referred to by the ISO standards [83], or punctual light sources, such light-emitting-diodes (LED) or lasers, often collimated and filtered. More comprehensive analysis is obtained distinguishing between the sagittal and tangential MTFs, using targets with different orientations [57]. Other types of test include the 1951 United States Air Force (USAF) and Snellen charts, while other cases have used the Sloan letters instead [41].

One of the earliest physical models for single-pass measurements was used by Oshika and Shiokawa [1], in order to assess the effect of soft acrylic IOLs folding procedures on MTF and resolving power [7]. The model eye was based on Gullstrand’s exact schematic eye. The MTF and resolving power were measured under veiling glare light.

**Figure 3:**
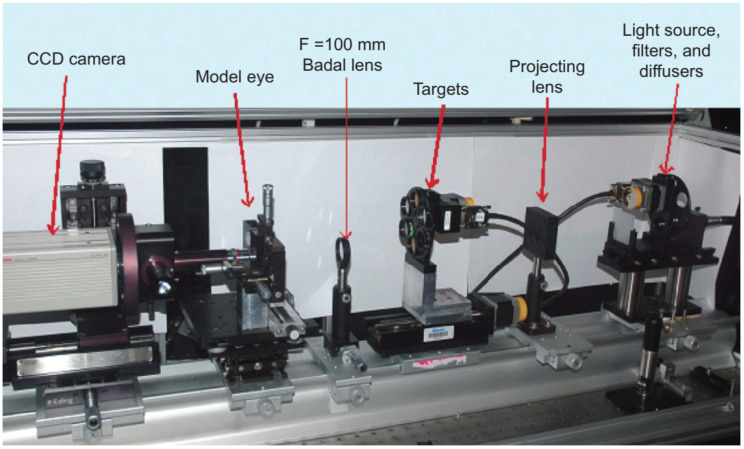
Typical setup of the ISO 11979–2 optical bench setup to evaluate the optical quality of IOLs, using a wet-cell eye model, and a Badal optometer (optional). Clinical Ophthalmology 2014:8 2105–2113 ([50]). Originally published by and reprinted with permission from Dove Medical Press Ltd.

**Figure 4:**
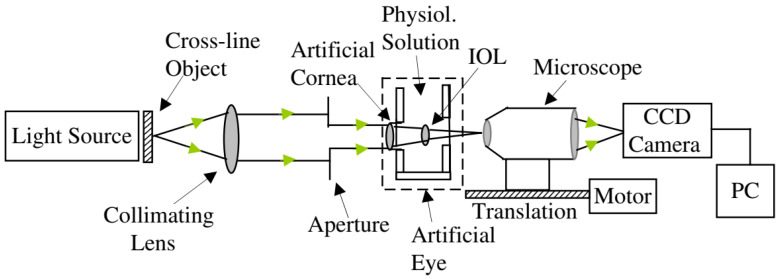
Schematic diagram of the eye model configuration used by Artigas et al, based on the ISO standards. Journal of Cataract and Refractive Surgery 2007, 33, 2111–2117 ([25]). Originally published by and reprinted with permission from Lippincott Williams & Wilkins, Inc.Norrby et al. [23] created and implemented one of their theoretical models as a physical device for MTF measurements (Figure 5). It seemed very simple and had the interesting characteristic of being packed into a very small enclosure, which could easily fit in different experimental setups. A CCD camera and suitable relay optics would simulate what the patient sees. The authors also mentioned the possibility of modifying it for wavefront measurements by using a diffusing retina, suggesting it could be used both for wavefront and single-pass measurements. Other single-pass models have been presented before as multi-modal cases [23,28]. Additional single-pass models for image formation will be presented in a later section dedicated to human vision.

The vast majority of single-pass eye models have been used in bench measurements to evaluate IOLs, using the 1999 ISO specifications described before [83]. They can follow this standard strictly [14,18,19,25,29,43,47,49,60,65], or implement slight modifications, namely by using modified corneas to better represent the human cornea or simulate various aberration conditions [13,27,29,31,33,43,50,56,68,71]. Indeed, the most recent iteration of the ISO standard (2014) introduced a new cornea with similar modifications [85]. Figure 3 presents a typical optical bench setup using a wet-cell model to evaluate IOLs ([50]). A microscope is often used to magnify the image at a transparent retinal surface and forward it to a CCD used to produce the retinal images. Figure 4 presents a typical schematic representation of such a configuration ([25]).

**Figure 5:**
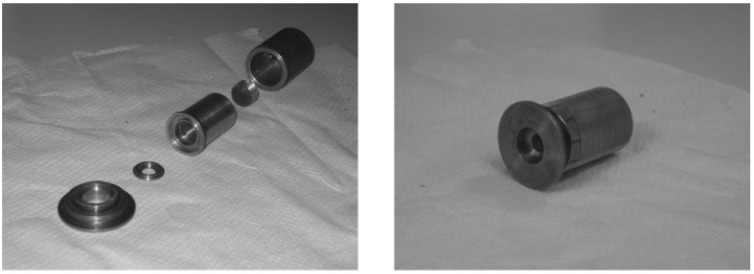
Photograph of the device used by Norrby et al.: (**Left**) individual parts; (**Right**) fully assembled device. An interesting feature of this eye model is the fact it is all its components are compacted into a small enclosure, whose size resembles a real eye. Applied Optics 2007, 46, 6595–6605 ([23]). Originally published by and reprinted with permission from Optica Publishing Group.

There are also single-pass techniques that do not involve the formation of a retinal image. Sheehy et al. [76] described in a patent application an artificial human eye, around 6.5 times the size of a natural eye, for testing the effectiveness of protective eyewear against harmful radiation (e.g., laser, ultraviolet rays, etc.). A transparent or translucent image plane functioned as the retina, which could be connected to a CCD, an optometer, a radiometer, etc. Radiation intensity could then be quantified, by analyzing pixel intensity, radiation levels, etc.

Regal et al. [70] developed an optomechanical eye model phantom by combining optical light propagation and thermal characteristics, to provide a system for accurately modeling the effects of laser surgery. This model was mainly composed of a main body and ancillary optical components, and a tunable microfluidic iris, and was fabricated using a 3D printed holder and modified polydimethylsiloxane. It also included a cornea, lens, ciliary body, sclera, and phosphate buffered saline (BSS) emulating aqueous and vitreous humors. A LED light source was mounted in the stand 1 mm in front of the cornea and directed into the iris for optical single-pass measurements. A concave depression in the center of the design simulated the retina at the back of the eye, where a photodiode was located for single-pass optical measurements performed for different angles, to compute the transmissibility of the pupil. Thermal measurements were also performed and will be detailed later in a dedicated sub-section.

Other single-pass techniques involve laser ray-tracing (LRT). Such an example is presented by Moreno-Barriuso et al. [9]. Another case is presented by Barbero et al. [16], where a model with a camera objective as the cornea and a diffuser surface was placed at the focal plane, acting as the retina. Aerial images were captured by a camera for a ray optics analysis. To avoid speckle noise in the aerial images, laser exposure times were increased while the diffuser was moved vertically. Wave aberrations were computed from the images. Eppig et al. [36] used single-pass ray-tracing to assess the effect of decentration and tilt on the image quality of aspheric IOL designs in a model eye. The model was based on the Liou-Brennan model eye [93], and included a pupil decentered nasally by 0.50 mm, resulting in a visual axis, which is tilted by 5 degrees (“kappa”) relative to the optical axis. The model also incorporated slight crystalline lens decentration (approximately 0.50 mm temporally) and tilt (<5 degrees relative to the line of sight), which showed good agreement with previous phakometric measurements. The wet-cell-based model of Terwee et al. [27] also included a simple qualitative ray-tracing analysis, by making the light path visible in the liquid behind the lens by adding 0.1 g/L of milk powder. Images were then taken with a digital camera through a surgical microscope and the presence of a near and far focus was assessed.

Of course, even single-pass eye models have their own limitations. The MTF and PSF techniques assume the eye as a linear optical system, and the retina is not spatially linear. A sensor such as a CCD or a CMOS assumes a uniform density along the entire sensing surface. This should be fine whenever the measurements are applied at the fovea region where the density of photoreceptors is the highest. On the other hand, in peripheral regions this linearity is lost and the image quality degrades with eccentricity.

### 3.4. Fundus Imaging and Retinoscopy

**Figure 6:**
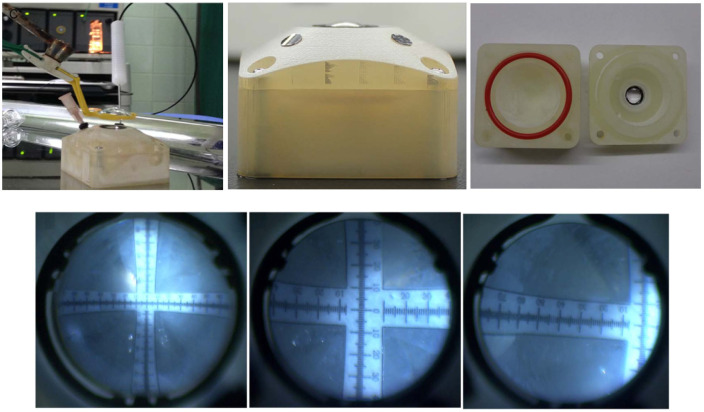
(**Top**) Detailed photographs of Xie et al. eye model. (**Bottom**) Photographs of fundus pictures of the angle bars photographed under 128D lens, 60D lens, and 60D lens imaged through the eye model. PLoS ONE 2014, 9, e109373 ([52]). Originally published by and reprinted with permission from PLoS ONE.

In this section, the eye models for measurements such as fundus ophthalmoscopy and retinoscopy are included. These methods are based in the observation of the retinal surface illuminated by external light. Despite the light passing twice through the optics of the eye, only the light coming from the illuminated retina provides information about its optics. Therefore, these techniques are not exactly double-pass. Rudnicka et al. [6] used their eye model to investigate the relationship between the actual size of a fundus feature and its photographic image in two different fundus cameras using fundus photography. The model presented in Inoue et al. [38] glued a 1951 United States Air Force (USAF) test target to the posterior surface of a model eye, based on Gullstrand’s model [1], and then the model eye was filled with balanced salt solution (BSS). The contrasts of the gratings were evaluated under endo-illumination. Similarly, Xie et al. [52] 3D printed an eye model based on Navarro’s eye [92], and glued angle scale bars from posterior to periphery of the posterior surface of the enclosure, filled with BSS, for fundus viewing studies (Figure 6). In the eyes developed by Fung et al. [53] and Yusuf et al. [58], a spherical model based on Gullstrand’s model of the human eye was used to evaluate fundal field-of-view of the lens of different equipment of ultra-wide field retinal imaging, such as ultra-wide field fundus fluorescein angiography. The posterior internal surface was marked with reference grids and infrared reflectance images of the. In the eye model by Winter et al. [63], an on-axis aligned model eye was used to measure the transverse chromatic aberration (TCA) across the visual field of the human eye objectively, using an adaptive optics scanning laser ophthalmoscopy (AOSLO) system using infrared (IR) light. These measurements were also performed in a second-pass setup. The model of Arell and Kolari previously mentioned [4] was used as an object in direct and indirect ophthalmoscopy where the retina was a slide with a picture of an arrow. The retina could also be back-illuminated, to make observation easier.

### 3.5. Scheimpflug and Purkinje Imaging

**Figure 7:**
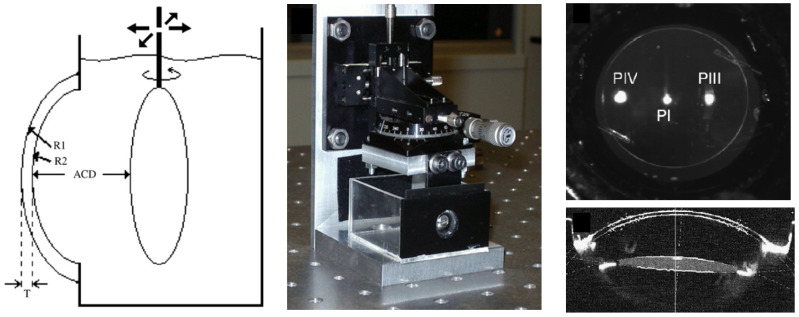
(**Left**) Scheme depicting the device used by De Castro et al. R1, R2 and T are the anterior/posterior radii and thickness of the artificial cornea. ACD is the anterior chamber depth. The arrows show that the IOL can be translated and rotated; (**Center**) photograph of the fully mounted device used by De Castro; (**Right**) ray images acquired from the Purkinje (**top**) and Scheimpflug (**bottom**) systems used by De Castro at al. Journal of Cataract and Refractive Surgery 2007, 33, 418–429 ([22]). Originally published by and reprinted with permission from Wolters Kluwer Health Inc.

The use of artificial model eyes to evaluate Scheimpflug and Purkinje modalities is way simpler than for other applications, as these models do not need to include the posterior segment of the eye, i.e., the vitreous chamber and the retina. Dubbelman and Van der Heijde [11] developed a simple physical eye model to validate a procedure that used Scheimpflug imaging to evaluate the crystalline lens in aging humans. The device included a cornea, and a biconvex lens immersed in water to work as the crystalline lens, which could also be translated relatively to the cornea. De Castro et al. [22] used a similar device to measure tilt and decentration of IOLs using Scheimpflug and Purkinje imaging systems. The IOLs would be placed at the position of the crystalline lens, and could be translated and rotated to simulate various in vivo conditions. Figure 7 shows a schematic representation of the device used by De Castro, a photograph of it and images acquired from both modalities from the same device. Another reference to Purkinje image measurements using eye models is described in Barry et al. [12], where six physical eye models were built for ophtalmo-phakometric measurements of ocular radius and curvature. The eye models were based on Le Grand’s schematic eye [94], with varying combinations of corneal and crystalline lens parameters, and the design was previously described In [95]. Briefly, each model included a cornea and a crystalline lens, mounted on a brass body of four segments that formed an enclosure which was filled with distilled water.

### 3.6. Optical Biometry and OCT

In this sub-section, eye models used for dimensional measurements, such as biometry and OCT imaging, are discussed. Despite Xie et al. [52] using biometry and OCT measurements in their eye device, these were only for characterization purposes, and will not be detailed further.

**Figure 8:**
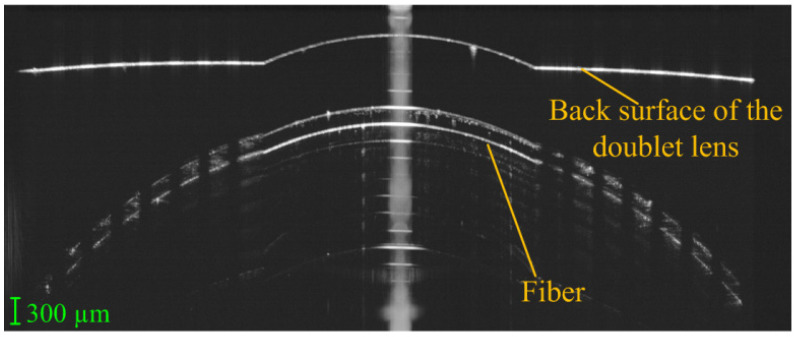
OCT B-scan acquired using the eye model of Wang et al. Journal of Innovative Optical Health Sciences 2021, 14, 2150010 ([72]). Originally published by and reprinted with permission fromWorld Scientific.

The only physical model found to evaluate optical coherence tomography (OCT) measurements was described by Wang et al. [72], which developed a tool for retinal OCT instrument calibration. The model comprised a doublet lens, a single filament, a piece of glass plate and a microsphere-embedded phantom. The rear surface was marked with concentric circles and served as a retina to measure the angular field-of-view (FOV). An empty chamber between the small plane of the second lens and the first surface of the glass plate was used to measure the depth scaling of the OCT, while the small flat surface on the peak of the second lens was used to test the signal to noise ratio (SNR). The single filament with 125 µm diameter was used to check the co-alignment of preview and OCT scan. The phantom with microspheres could test both the lateral and axial resolution. Figure 8 presents an OCT B-scan acquired using the eye model.

Regarding biometry applications, some examples were found. Birkner et al. [39] created an eye model to evaluate biometric measurements inside the model eye using a two-wavelength Fourier domain low-coherence interferometer. The test subject was a model eye, including a rigid polymethylmethacrylate (PMMA) contact lens (CL) as the cornea, a commercial biconvex lens as the crystalline lens, and a reflecting surface as the retina. The cornea–lens and cornea–retina distances could be adjusted. The device was filled with water. The refractive index of the bulbus was calculated using the known length and refractive index of the crystalline lens and the measured optical length of the bulbus without the lens. A similar eye model was used by Al-Mohamedi et al. [67] to compare and evaluate three different swept-source interferometers for eye length biometry. It also had a PMMA hard CL cornea, an IOL as the crystalline lens, and a diffuser as the retina. The interior was filled with water. Mao et al. [61] used a model eye (OEMI-7, Ocular1 Instruments Inc., Bellevue, WA, USA) to validate OCT full eye biometry. The model eye was made of PMMA, and the model crystalline lens was replaced with the IOL. Saline solution was used to fill the model’s anterior chamber and vitreous chamber. The enclosure seemed to be painted to make it opaque.

### 3.7. Other Modalities

In this sub-section, the cases that did not fit in any of the categories previously discussed, but which were still interesting enough to include in this review, are discussed.

Physical eye models have also been developed for radiation safety studies. Sheehy et al. [76] described one such device in a patent invention, as seen previously. Trinavarat and Atchaneeyasakul [10] created an eye model to simulate the condition of foldable IOLs in the capsular bag and evaluate laser damage in order to choose appropriate laser energy levels for posterior capsulotomy. Each IOL was sandwiched between two rubber membranes, to act as the posterior capsule and anterior capsulotomy. The model was then submerged in a water-filled chamber. The front part of the chamber had a transparent aperture similar to a very archaic cornea through which a laser beam would be applied. The laser used was a Nd:YAG laser. As an interesting fact, this is the only eye model found to consider the lens capsule.

Alvarado et al. [55] created a tunable opto-mechatronic system to mimic the focusing and regulation of illumination in the formation of images made by the human eye. The main features of this system were the automatic control of the pupil size and crystalline lens power, in a biomimetic fashion. However, what distinguishes this model is the fact that it could adapt the pupil size from the intensity of incident light. A solid elastic lens (SEL) made of polydimethylsiloxane (PDMS) mimicked the crystalline lens, with parameters based on one of Navarro’s eye models [96]. An automatic diaphragm simulated the iris of the human eye and a light sensor made it possible to control the diaphragm through a control software module. The SEL was also controlled electronically. A characterization of the system was performed with standard values of luminosity of the human eye, to calibrate and to validate the entrance illumination levels to the overall optical system, though the diaphragm. A finite element modeling and mechanical analysis of the system controlling the SEL were also performed. Despite it is not explicitly stated, a CCD camera could also be included for single-pass measurements (as shown in a scheme of the device). As a side note, the authors of this review cannot be sure if this system was actually constructed, or just computationally modeled. For that reason, it was included in this subsection.

Finally, as stated before, Regal et al. [70] also performed thermal measurements with their eye model. This was done using an 850 nm continuous wave infrared laser diode and a thermal camera installed on the test bench, pointing to the back of the physical model. Thermal measurements were performed on the retina surface and on the back of the ciliary body. For these measurements, the vitreous chamber was filled only with air, as it was not possible to include the vitreous humor since the main structure had to be removed for the ciliary body measurements.

## 4. Eye Models for Human Vision

In the previous sub-sections, several single-pass models were presented, most of them able to simulate a retinal image under different optical conditions. In this section, a sub-class of such models is presented that includes cases particularly relevant for the field of human vision simulation. The devices presented here will be discussed in additional detail.

The eye has to detect and resolve objects in the visual field, and different parts of the retina are responsible for that. The human visual system capacity depends strongly on environmental conditions. As such, it is of interest to artificially simulate various situations our visual system faces, such as our ability to recognize and track objects under different illumination conditions. Here, dynamics such as accommodation, which affects the quality of the image obtained, or the pupil diameter, which affects the illuminance, have a high impact in image formation, as they determine the contrast and brightness of the features present in the image.

In addition, beyond a static centered image formation system capable of recording the on-axis image for posterior analysis, which is already provided by several physical eye models, it should be possible to record images off-axis in order to provide a complete field of view. While the central part of the retina is able to resolve fine details and perceive colors, peripheral areas beyond the 20 degrees of the visual field are good at detecting moving objects, blinking lights, or contrast thresholds, but not at recognizing shapes, resolving details or perceiving colors. Unfortunately, off-axis features are still a rarity in physical eye models.

**Figure 9:**
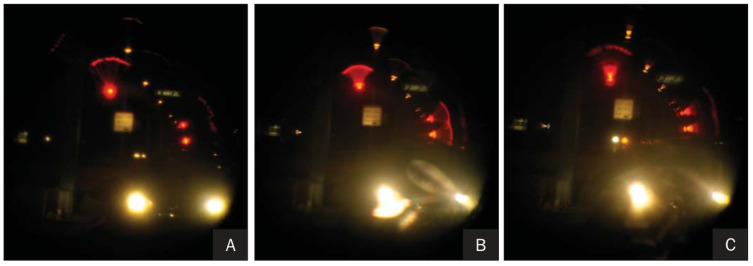
Night driving scene through three different multifocal IOLs, imaged through the eye model presented by Choi at al.: (**A**) ReSTOR, (**B**) ReZoom, and (**C**) Tecnis ZM900 multifocal IOLs. Journal of Refractive Surgery 2008;24(3):218–222 ([26]). Originally published by and reprinted with permission from SLACK Incorporated.

One significant challenge for the visual system occurs under night driving conditions when the pupil aperture is more dilated and the system exposed to stronger optical aberrations. It is also common for the driver to face bright sources of light that can easily temporarily blind a part of the visual field. These challenges are more critical in drivers with pathological conditions, wearing corrective optical devices or undergone surgical procedures. Choi and Schwiegerling [26] used a wet-cell-based eye model to measure the optical performance of some specific IOLs, and simulate night driving conditions. The artificial eye had both the average spherical aberration and chromatic aberration levels found in the human eye. The illumination system consisted of a white light source and a condenser lens, and a filter wheel with multiple narrow pass filters was inserted into the illumination path to select various wavelength bands across the visible spectrum. A narrow slit was mounted on a translation stage and placed at the front focal plane of a Badal lens. A CCD camera captured the image produced by the eye system. The polychromatic MTF for the lenses was calculated from the images by examining the blurring of the slit beam. In addition to that, the model eye was also used as a portable device to photograph nighttime driving scenes for a qualitative evaluation, as seen in Figure 9.

Alba-Bueno et al. [66] used a wet-cell configuration based on the ISO specifications to examine and assess the halos generated in distance vision by multifocal IOLs. To determine the halo diameter and its relative intensity, they used a pinhole illuminated by a narrow-band green LED. The artificial cornea used induced a similar amount of spherical aberration in the IOL plane as the average human cornea. The image of the pinhole formed in the plane of the distant focus of the eye model was captured with appropriate resolution by means of a CCD camera fitted to a microscope objective. Results were compared with psychophysical tests performed on subjects, using a glare source as input.

**Figure 10:**
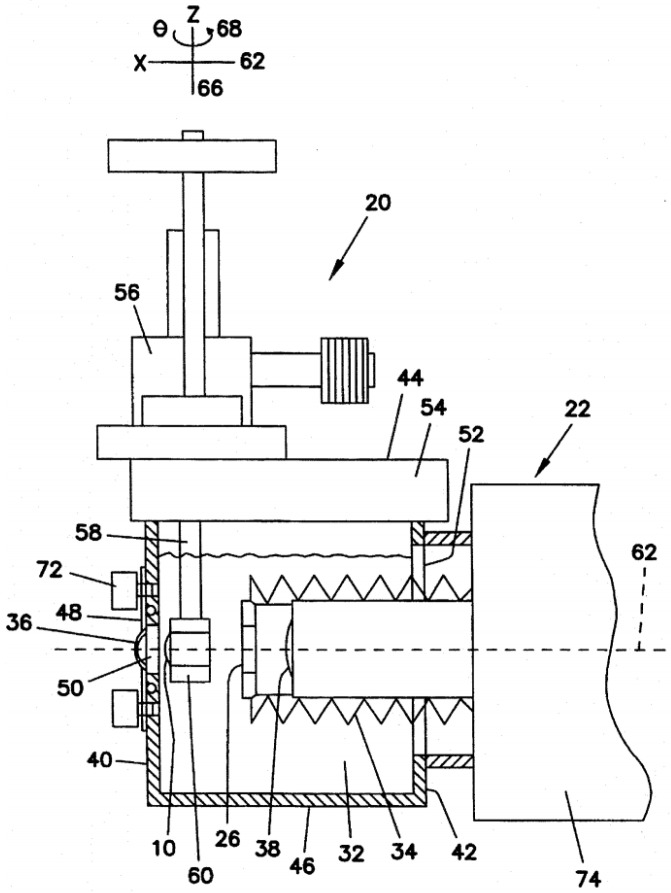
Drawings of the device described in Schneider and Keates. Originally published by and reprinted from U.S. Patent 5,652,640, 29 July 1997 ([74]).

Schneider and Keates [73,74] described in a patent an invention to evaluate on-axis vision through a bifocal IOL or a bifocal CL (Figure 10). It could also be used for research applications, by recording data to images and measuring optical parameters, and adapted to evaluate IOLs during production. The device had a cornea (which could be pressurized as in a human eye), which could also be a CL, an IOL holder as the pupil, and could be filed with any fluid, such as a saline solution. An attached fovea window protruded into the eye model, to project a fovea image to an external observer for evaluation. It could translate in two directions, allowing for off-axis visualization. The image from the fovea window could be projected onto a film, a video camera, or similar devices for storage and analysis of the image. With an appropriate lens or prism arrangement, such projection could occur simultaneously with a subject viewing the image at the fovea projector eyepiece.

Ohnuma et al. [75] reported in a patent an apparatus for simulating a retinal image obtained when an IOL was implanted in the eye, or when a CL or an eyeglass lens is worn on with the eye. One of the embodiments was similar to a simple wet-cell setup, where an IOL was placed for evaluation. The assembly had at least a front-end lens and a rear-end lens, which were glass plates that created a chamber capable of holding liquid. It also had an aperture attached in front of the IOL. Anterior to the wet-cell, a photographic lens assembly was used to allow a clear image to be displayed on the whole display unit. In another embodiment, the cornea had a realistic shape, and a CL could be placed on top of it. Distilled water or other liquid was dropped between the cornea and the CL to simulate tears. It also included a pupil and a posterior multiple lenses system to simulate the crystalline lens, but details were not provided about it. The sensor was a CCD or other imaging system. Fovea images of object would be captured by the camera, and then be evaluated.

**Figure 11:**
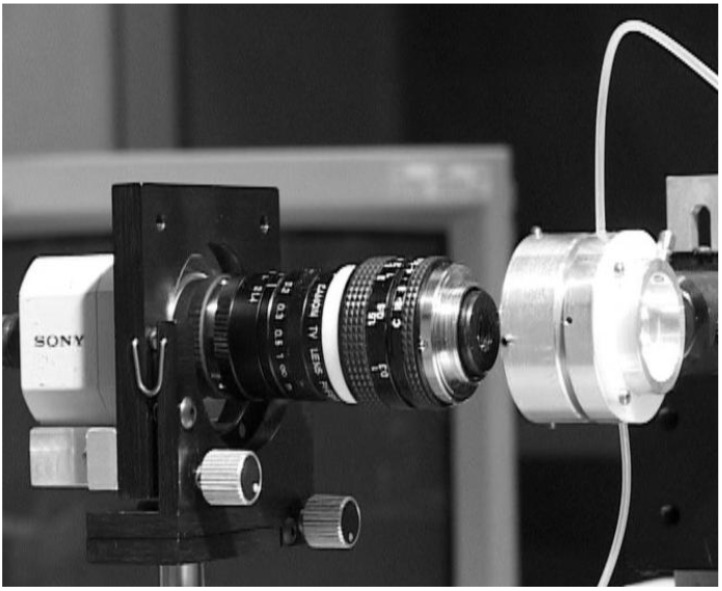
Photograph of the device used by Gobbi et al. In the experimental setup, the fully assembled eye model is on the right side of a camera objective. A cornea lens can be easily seen attached on the right surface of the cylindrical model. Proceedings of the Ophthalmic Technologies XIII; SPIE, Bellingham, WA, 2003; Volume 4951, p. 104 ([97]). Originally published by and reprinted with permission from Gobbi, P.G.; Fasce, F.; Bozza, S.; Brancato, and SPIE.

**Figure 12:**
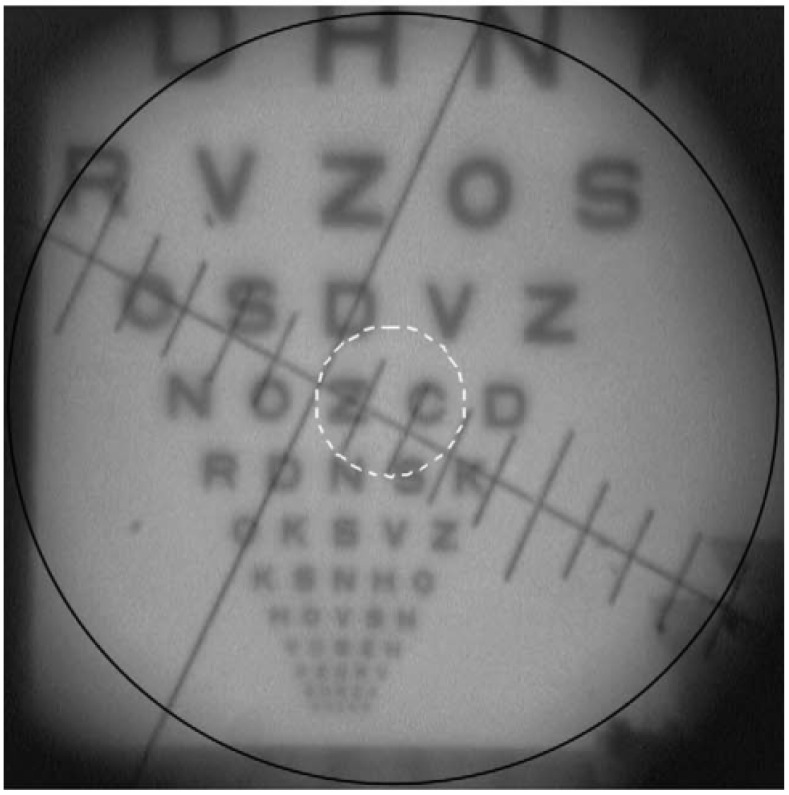
The imaging performance of the optomechanical eye model presented by Gobbi et al., showing the digitized electronic record of the retinal image corresponding to the projection of the ETDRS chart. Journal of Cataract & Refractive Surgery 2006, 32, 643–651 ([20]). Originally published by and reprinted with permission from Wolters Kluwer Health Inc.

Gobbi et al. [20] developed a small packed device to provide data on the visual performance with monofocal IOLs, for objective comparison between IOL models and direct correlation with the relative visual performance attainable in vivo. This model was first described in a work to characterize the imaging properties of multifocal intraocular lenses [97], but later re-published with additional details (Figure 11). The retina was modeled by a lens plate (ocular fundus) with a graduated reticle on its inner surface, connected to an optical relay using television objectives to magnify the image and forward it to a CCD. The device was used to simulate psychophysical tests usually performed on humans, such as visual acuity and contrast sensitivity tests at far and near distance. The investigators took digitized samples of the image produced by the CCD camera, as well as readings given by a masked subject looking at the same image on a television screen and asked to identify letters on visual acuity charts and grating orientation in contrast sensitivity patterns. Figure 12. visually depicts the imaging performance of the optomechanical eye model, showing the digitized electronic record of the retinal image corresponding to the projection of an ETDRS chart.

Barcik et al. [30] designed an anatomically and optically equivalent bench-top model eye, representative of the average human adult eye and suitable for testing the on- and off-axis performance of contact, spectacle and intraocular lenses. The device resembles a wet-cell setup, but has an opaque enclosure and seems portable enough. A rear glass window connected to a CCD camera through a microscope emulated the retina. A contrast analysis of image features was performed, including Siemens stars, edge images, and sinusoidal patterns. The MTF was estimated as the ratio of Michelson contrast in the image of sinusoidal luminance distribution to the contrast in the said distribution itself.

**Figure 13:**
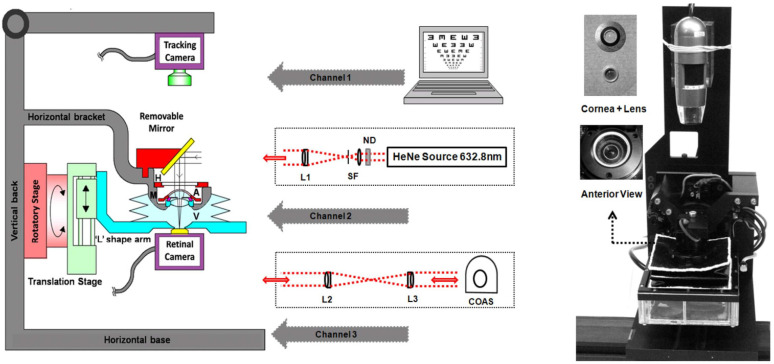
(**Left**) Schematic painting of the artificial eye used by Barakraju et al.; (**Right**) photograph of the device. A magnified overview of the anterior chamber of the model eye shows the humidity chamber, cornea, and sclera. The individual cornea and crystalline lens are also shown. Optics Express 2010, 18, 16868–16882 ([37]). Originally published by and reprinted with permission from Optica Publishing Group.

One of the most complete eye models so far in terms of features and measurement modalities was built by Bakaraju et al. [37], being representative of the average human adult eye and suitable for testing the on- and off-axis performance of contact, spectacle and intraocular lenses. A follow-up patent by Ehrmann et al. [80] reported some additional features, such as the retina including a photoemitter for second-pass measurements, or the use of optic-fibers in a similar fashion as Arianpour et al. [46], while also mentioning a wide range of potential applications, such as the evaluation of corneal re-shaping and corrective lenses, as well as to calibrate ophthalmic instruments, or to simulate functions, defects, pathologies, surgical modification and/or injuries of the natural eye. Applications of the model were later reported in [98]. The model had a cornea based on the Gullstrand model [1], where a contact lens could be placed on top (Figure 13). A special humidity chamber with temperature control made it possible to minimize evaporation of soft contact lenses. It also included a crystalline lens, a pupil, and a retinal surface. The anterior and posterior chambers were filled with de-ionized, purified water. A complementary metal-oxide-semiconductor (CMOS) sensor was used as the retina for image quality measurements, which could be rotated to cover the entire curved retinal surface. This made it possible to assess optical quality via a logMAR chart presented on a visual display unit positioned at a given distance from the model eye, or via single-pass measurements by analyzing in the Fourier domain a spot image from a laser source to obtain the OTF. A diffuse reflecting plate could also be used instead of the CMOS camera for double-pass experiments via an autorefractor or wavefront aberrometer positioned in front of the eye. The device also included a tracking camera unit to provide information about the fit, centration and meridional orientation of a test CL placed on the cornea.

**Figure 14:**
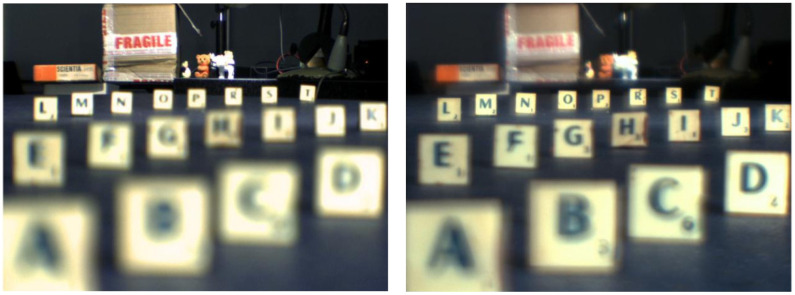
The 3D scene imaged by Petelczyc’s et al. artificial eye: ((**left**) uncompensated; (**right**) compensated by the LSOE). Optics Express 2011, 19, 25602–25616 ([42]). Originally published by and reprinted with permission from Optica Publishing Group.

Petelczyc et al. [42] built a human presbyopic eye model based on the Gullstrand parameterization [1] to analyze the imaging properties of the light sword optical element (LSOE) applied as a contact lens to the presbyopic human eye. The eye included a BK7 cornea, to where the LSOE was applied. Using the constructed model and the fabricated refractive structures, a 3D scene with objects and letters at different distances was imaged using a CCD camera (Figure 14). A monofocal corrective lens was also used on the model, for comparison with the LSOE.

Ohnuma et al. [40] created a model eye to compute retinal image contrast with combined correction of chromatic and spherical aberrations. It included an IOL and an artificial cornea with human ocular longitudinal chromatic aberrations and average human spherical aberrations. Landolt “C” optotypes were illuminated using a fluorescent light source, and images of them were obtained using two-dimensional luminance colorimeter camera. Image contrast was analyzed for each color image, in order to evaluate chromatic aberrations associated to each type of cones.

**Figure 15:**
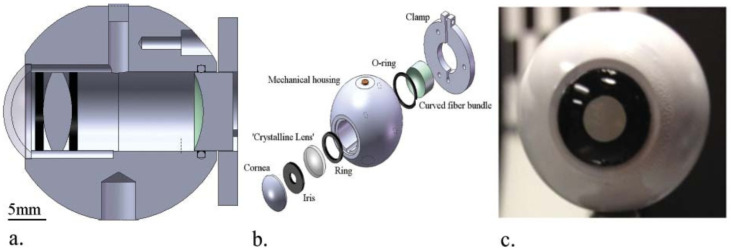
Eye model used by Arianpour et al. (**a**) Cross-sectional view of the optomechanical eye model. (**b**) Exploded view with the individual parts listed. (**c**) The assembled optomechanical eye model that is comparable to a life-size human eye. Journal of Refractive Surgery 2013;29(2):126–132 ([46]). Originally published by and reprinted with permission from SLACK Incorporated.

Arianpour et al. [46,99] created a low-cost optomechanical model eye for investigation of refractive errors in clinical and basic research studies (Figure 15). The model was also very detailed and included a cornea, a crystalline lens which could be replaced by an IOL, and an iris pupil. Device parameters were based on Navarro’s eye model [96]. The more interesting feature was a fiber-optic bundle connected to a CMOS with relay optics for image magnification, to simulate a curved retina. The device was characterized by measuring image sharpness of a slanted edge on a black and white chart, a standard technique for measuring the resolution of digital cameras. This also made it possible to compute the MTF, which was compared with a ZEMAX-simulated MTF. The device was then used to test IOLs at different distances, using images of the 1951 U.S. Air Force resolution chart as target. Real scene images were also acquired, for qualitative analysis.

**Figure 16:**
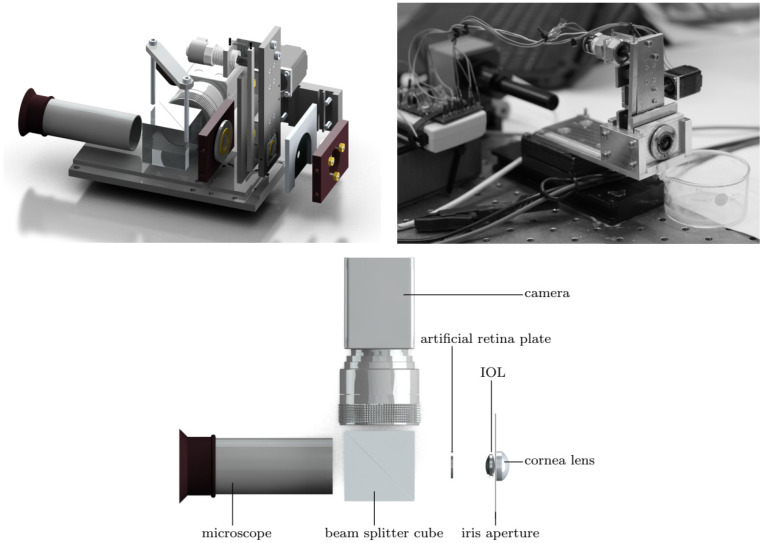
(**Top**) 3D rendering and real photography of the device used by Drauschke et al.; (**Bottom**) schematic painting of the measurement setup. © 2013 International Federation of Automatic Control. Reproduced with permission from Andreas Drauschke, Elisabet Rank, Lukas Traxler, Kirsten Lux, Christian Krutzler, “Mechanical Eye Model for Comparison of Optical and Physiological Imaging Properties”. IFAC Proceedings Volumes, 46/28 (2013), pp. 1–12 ([48]).

Drauschke et al. [48] created a mechanical eye model for comparison of optical and physiological imaging properties. It consisted of a cornea lens, an iris aperture, an IOL and an artificial retina. The space in between these components was filled with an artificial aqueous humor with the correct optical properties. The IOL could be tilted and decentered and a vibrational analysis was also performed to quantify displacement error or other related errors during the mounting of the IOL. A microscope was used to project the image from the artificial retina into the retina of a test person, for physiological image quality measurements. A beam splitter was positioned between the artificial retina and the microscope, allowing also simultaneous optical image quality measurements by placing a camera perpendicular to the optical axis of the microscope. A 3D representation and a real photography of the device, together with a schematic drawing of the measurement setup are in Figure 16. Measurements from ISO targets charts were used to compute metrics such as the spatial resolution, contrast, MTF, Strehl Ratio, etc., in order to characterize the system. A HS sensor allowed the measurement of wavefront deformations and Seidel or Zernike polynomials. The internal fluid was also characterized in terms of Sellmeier coefficients and dispersion.

**Figure 17:**
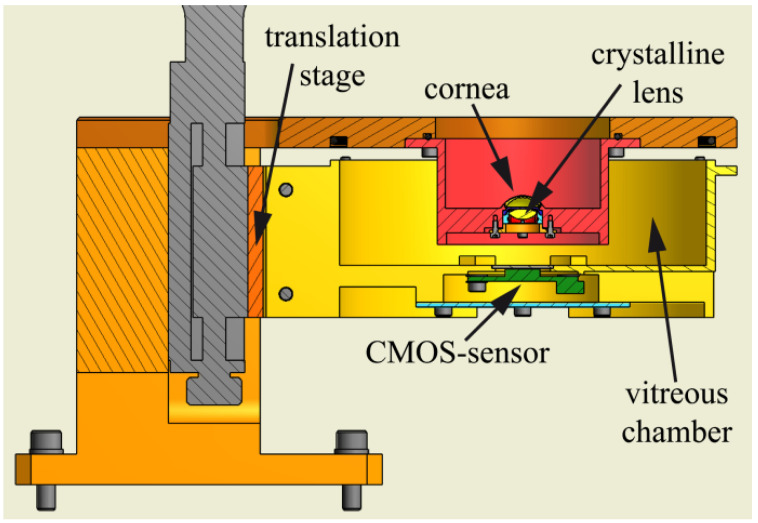
Sectional drawing of the model presented by Ackermann et al. Biomedical Optics Express 2013, 4, 220 ([45]). Originally published by and reprinted with permission from Optica Publishing Group.

Ackermann et al. [45] used a model eye to investigate optical side-effects of femtosecond-laser treatment in refractive surgery. This model had some features based on Bakaraju’s model [37], but seemed simpler and more compact. The artificial cornea was a contact lens used to simulate laser treatment, located within an indentation. A pupil and a crystalline lens were also present. The artificial vitreous chamber could be moved by means of a motorized translation stage, and its vertical configuration did not require the use of a bladder like enclosure (Figure 17). A color CMOS camera beneath a glass window on the bottom of model eye served as the artificial retina. All chambers were filled with distilled water. Rainbow glare was investigated by means of a high-power LED, with illuminance of 3.5 lux simulating high beams of an oncoming vehicle at night. Visual acuity was also investigated by means of Landolt “C” rings in order to evaluate contrast sensitivity, where a 400 W halogen floodlight was used as light source.

Some recent eye models started to introduce modern features such as electronic controllable pupils and crystalline lenses. Some of such models were already presented above ([55,57,70]), but were not relevant enough for human vision applications. Relevant models are briefly described below.

Liang et al. [51] developed a device based on the Gullstrand model [1], which consisted of a bionic cornea lens, a voice coil motor controlling a compression ring to compress a bionic crystalline lens, a substrate, and a CCD sensor. By controlling the current of the voice coil motor, the displacement of the compression ring could alter the curvature radius of the bionic crystalline lens, thus adjusting the focal length of the whole system. Images with text were captured under different displacement loads. The object distance was adjusted to get a clear image on the CCD sensor and the back focal length was measured from the CCD sensor plane to the lens frame surface facing the sensor.

**Figure 18:**
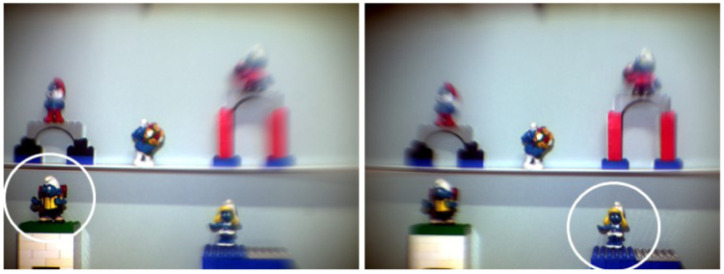
Simulation of saccades using the eye model of Förster et al. For each image, the pivot axes of the optical group is pointing in a different direction, so that for each individual image a different part of the scene is highly resolved (highlighted by a white circle. Optics Express 2015, 23, 929 ([54]). Originally published by and reprinted with permission from Optica Publishing Group.

Förster et al. [54] created an artificial eye to model accommodation and gaze movement. It used an aspherical surface and diffractive optical element to model the cornea and a tunable fluidic membrane crystalline lens, comprising an optical group and a CCD camera. To simulate saccadic movements, a mechanical Gimbal-mounted system was used for adjustment of the pivot axes of the optical group with respect to the camera, which remained fixed during the image capture, thus changing the focused object. An example of a saccade simulation is presented in Figure 18.

**Figure 19:**
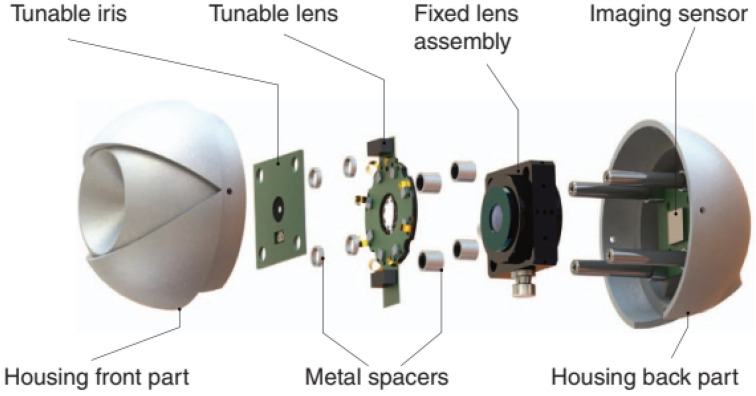
Exploded view of the engineered eyeball assembly constructed by Petsch et al. Light: Science and Applications 2016, 5, e16068 ([62]). Originally published by and re-printed with permission from Springer Nature.

**Figure 20:**
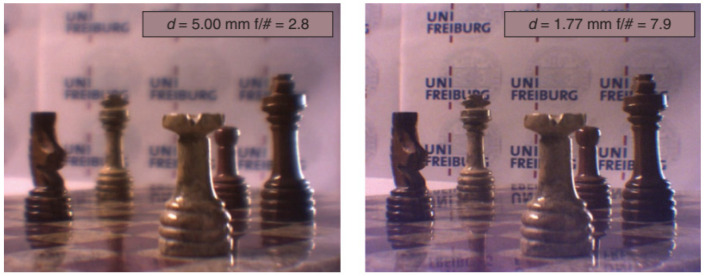
Scene captured by the model constructed by Petsch et al. for aperture tuning using the fluidic iris: variation in the measured DOF for iris diameters of 5.00 (**left**) and 1.77 mm (**right**). All figures and the background text are in focus for d = 1.77 mm. The distance between the front and rear figures is 220 mm. Light: Science and Applications 2016, 5, e16068 ([62]). Originally published by and reprinted with permission from Springer Nature.

Petsch et al. [62] devised another biomimetic tunable imaging system using soft-matter micro-optics. The model was mainly focused on the use of a tunable iris and a tunable crystalline lens, whose actuators would mimic the iris and ciliary muscles. It integrated a deformable elastomeric refractive structure whose shape was mechanically controlled through application of strain using liquid crystal elastomer (LCE) actuators; two forms of tunable iris, one based on optofluidics and the other on LCEs with embedded heaters; a fixed lens arrangement; and a commercial imaging sensor chip. A Cooke triplet replaced the cornea, and a CMOS sensor represented the retina. According to the authors, this device represented the first fully functional, soft-matter-based tunable single-aperture eye-like imager. An exploded view of the device is in Figure 19. The characterization of the imaging performance comprised measurement of MTF, sharpness, depth of field (DOF), focus position as well as qualitatively evaluable images as a function of tuning behavior (Figure 20).

Gu et al. [69] built an eye model with a very realistic retina. It included a front lens, an aperture, an aluminum shell sclera, and a hemispherical perovskite nanowire array retina, connected to liquid-metal nerve fibers to simulate the optic nerve. An ionic liquid humor filled its interior. This eye model was capable of acquiring images with resolution higher than a human eye.

## 5. Components of a Physical Eye Model

Despite the existence of several variations, a typical eye model has an anterior lens system that represents the cornea, an opaque barrier (usually circular) with an aperture to simulate the iris and the pupil, an interior lens to represent the crystalline lens, and a posterior viewing system which corresponds to the retina. A large number of models do not have a crystalline lens, but an IOL instead, either to mimic the crystalline lens, or for IOL characterization. Components may be static or dynamic, the latter allowing changes in their properties without the need for replacement. However, most devices opt for the former option. Some eye models also allow the relative distances between components to be changed. Most devices form open or enclosed chambers that may be filled with fluid to model the aqueous and vitreous humors.

Of course, other components that follow the anatomy of the human eye to a lesser degree may also be present in artificial eye models. Some include complex lens systems to model a single eye anatomic structure [19,62,75], while others tend to simplify and model multiple anatomic structures using a single lens [21,34,59] when additional complexity is not needed. Components that do not belong to the eye anatomy can also be present. For example, refs. [9,24,78] used aberrating plates to statically reproduce aberrations, while [17,21] used flexible biomorph mirrors to dynamically reproduce aberrations. Other models might use spatial and phase light modulators (SLM) to control the amount of incident light and add aberration. While not directly following the anatomy of a real eye, these components can be used to help simulating certain physiological functional features of a real human eye or eyes with disease or undergoing surgical interventions.

In an eye model, we can distinguish between static and dynamic components. A fully static eye model essentially comprises an aperture acting as a pupil, a system of two lenses acting as the cornea and crystalline lens, and a diffuser or sensor acting as the retina. On the other hand, a dynamic eye model has time-varying components, usually the pupil and/or crystalline lens. For example, an SLM can be used to act as the pupil and also shape the phase of the wavefront to simulate different image quality conditions, while the crystalline lens can be an optoelectronic lens able to accurately change its optical power to simulate shifting between near and distance focus of the human eye. Several static and dynamic components will be discussed in the following sub-sections.

### 5.1. Cornea

The cornea is the structure that provides most of the optical power to the eye. In humans, the normal cornea has a refractive index of 1.376 at 0.589 μm [92], an average anterior radius of curvature around 7.80 mm, a posterior radius around 6.5 mm, a thickness ranging between 0.5 and 0.6 mm, and asphericity conic constants ranging from −0.15 to −0.30 for both surfaces [100,101,102,103,104].

The cornea can be modeled by a lens, typically made of polymethylmethacrylate PMMA (acrylic), or fused silica, with refractive indexes ranging between 1.4 and 1.5, which are higher than the human cornea. A notable exception is the one presented by Bakaraju et al. [37], whose fluoropolymer cornea achieved exactly 1.376, the same value used in Gullstrand’s model [1]. Glass corneas (e.g., BK7, with a refractive index close to 1.5) have also been used [23,35] for increased power, but these usually had a more spherical aberration compared to PMMA [23]. Other materials have also been reported, such as PDMS [70] and Boston XO [32], while some patents have also suggested additional materials, such as silicone, silastic, polycarbonate, polystyrene, styrene acrylonitrile (SAN), topaz, cyclo-olefin polymers, CR-39, amorphous polyolefin, etc. [73,74,77]. One of the first cornea models was made of two thin glass shells, the space between filled with a liquid of index 1.377 [3]. There have also been reports where rigid and scleral contact lenses (CLs) were used as cornea models [11,39,45,67], while soft CLs were also mentioned [45].

The shape of the cornea lens depends on the intended application, the material used and the surrounding material. Most authors try to replicate the anatomical function to a certain extent, and thus choose generally a convex-concave meniscus lens, as in the Gullstrand model [1]. The curvature radius varies between applications, but is typically close to 7.7 mm–7.8 mm for the anterior surface and 6.4 mm–7.5 mm for the posterior surface, while the thickness is close to 0.5 mm in most cases, allowing a good approximation to the real eye case. However, other thicknesses are possible. For example, in [46] a thickness of 1.0 mm was used with the goal of improving the durability of the lens, without affecting significantly the quality of the images.

Other corneal shapes have also been reported by several authors [16,27,31,54,56], who have used double-convex (biconvex) lenses. Single surface lenses have also been used, with convex-plano lenses being very common [15,29,33,34,35,40,63,64,72,85], due to the fact that the human posterior cornea has a relatively small contribution to the overall performance, essentially due to the small difference in refractive index between the aqueous fluid and the cornea, and the difficulty in replicating the posterior cornea [64]. The anterior radius in these cases is usually larger, and quite different values have been used, with extremes ranging from 7.75 mm to 24.224 mm having been found. The thickness is also usually larger and more variable, with values of 4.5 mm having been reported. These lenses also usually require using a matching refracting index liquid in order to disguise the planar surface, imposing some restrictions on the mediums used, which can be a limitation when controlling aberrations, especially chromatic [64]. On the other hand, plano-convex lenses are not so common, but at least one has been found [42], with a posterior radius of 16.5 mm and a thickness of 2 mm.

Less anatomically accurate corneas also exist. For instance, the model presented in [4] used a single zoom-objective lens as the entire optics of the device, the system described in [21] used a lens with a focal length of 25 mm, simulating both the crystalline lens and the cornea, andthe device in [59] consisted of a single lens that was 50 mm in focal length. Compound lenses were also used, for example, ref. [62] used a Cooke triplet, while [19] used a biconvex + concave–planar doublet. It is rare to find eye models with no cornea, but some cases do indeed exist, such as [57], where the purposes of the eye models built did not require a functional cornea.

The cornea is usually designed to have the minimal possible amount of spherical aberration, or a specific amount of such aberration. As per the 1999 ISO 11979-2 recommendations for IOL evaluation [83], an aspheric (spherical aberration-free) achromat doublet lens (biconvex + concave-plano) has been used, and eye models based on these standards usually follow these specifications. However, as we have already seen, a lot of cases try to approximate the actual asphericity of the average human eye, and the ISO cornea model is often modified to introduce aberration. The most recent version of the ISO standard (2014) also followed this need, and introduced an alternative convexo-planar more realistic cornea [85].

Custom corneas may be modified as well. The anterior surface is often polished to achieve a prolate surface and its resultant aberrations, with conic constants ranging between −0.5 and 0 having been seen. The posterior surface is usually kept spherical. However, this is not always the case, as spherical and oblate anterior surfaces have been used [29,30,35,40] and aspheric posterior surfaces as well [37]. Typically, induced spherical aberration (SA) amounts vary between around 0.1 and 0.3 µm (RMS) for the standard 6 mm entrance pupil, which is in the physiological range. In other cases, the cornea is aberration free, as in the 1999 ISO reference, in these cases the SA values are close to zero. Other aberrations such as coma and astigmatism are usually measured and quantified rather than quantitatively modeled in the cornea design.

In most devices, the cornea actually corresponds to the anterior wall of the eye model, effectively sealing it from the external environment. However, some models used corneas at different locations. For example, refs [24,62] used their cornea lenses after the tunable lenses representing the crystalline lens. As for mounting, the cornea may be attached to the bulk of the device using special holders, boreholes, joints, etc., as will be discussed later. Some configurations make it possible to position a CL on top of the cornea [37,38,45,75,77,80]. According to [45], an anterior radius of 8.6 mm for the cornea is the most appropriate to add a standard daily wear CL. In these cases, it may be advantageous to coat the surface of the cornea with a tear film made of drops of water, saline, or natural tears [75,77]. To prevent evaporation of soft CLs, the cornea lens system [37,45,80] or the entire device [77] may be placed inside a controlled temperature/humidity chamber.

Other modifications may also be applied to the cornea. For example, ref. [79] coated the cornea lens with a dopant such as boron nitride in order to approximate the light scatter normally found in a human cornea.

### 5.2. Crystalline Lens

The natural crystalline lens in the human eye has a graded refractive index ranging from 1.38 to 1.43 [46], but this is not usually incorporated in eye models. It is a biconvex lens with an anterior radius of curvature between around 9.0 and 11.5 mm, a posterior radius varying between −7.0 mm and −6.0 mm, and a thickness between around 3.5 mm and 5.0 mm, depending on age and accommodation [103,104,105,106,107]. According to [105], the mean change in lens thickness for up to a 5D accommodative stimulus ranges from 0.01 to 0.26 mm. Relatively to asphericities, ref. [107] found an anterior conic value close to +3.0 and a posterior conic value close to −1.5.

In the real eye, the main source of variation for defocus and spherical aberration is the fluctuation of the crystalline lens, whose power continuously changes within small ranges [24]. For this reason, there is a great interest in modeling different states of the crystalline lens. We have previously seen that biomorph mirrors and aberrating plates may be used for this purpose, but they do not represent the anatomic structure of the human eye. A more realistic way is using dynamic crystalline lens, but in most cases, static lenses are used instead.

Similarly to the cornea, static crystalline lenses may be made of PMMA, fused silica, hydrogel, silicone, Boston EX [45], or another polymer [37,108]. Dimensions and refractive indexes are usually in the physiological range, while asphericities can vary significantly between models and be less realistic. For example, ref. [46] used an anterior radius of 10.20 mm, a posterior radius of −6.00 mm, a thickness of 4.00 mm and a uniform refractive index of 1.420. In [64] a symmetric biconvex lens of 11.7 mm radius, a thickness of 3.6 mm, and a refractive index of 1.517 at 589 nm, were used. The eye model presented in [37] used two lenses with anterior radii between around 7 and 12 mm and posterior radii close to −6.3/−6.0 mm, both dependent on accommodation. The anterior asphericities were also dependent on accommodation and were between −5.0 and −4.5, while the posterior asphericities where −2.50 and −2.75. Refractive indexes were 1.429 and 1.423. The thicknesses were also dependent on accommodation and close to 3.6–3.8 mm.

An aspheric surface design was made to minimize spherical aberrations in [46], or help achieve a specific amount of spherical aberration in [38].

While the crystalline lens has a spatially varying refractive index, most artificial lenses ignore this characteristic due to its high complexity and little improvement in the image quality in static conditions [46], and thus are fabricated with a uniform index of refraction optimized for image quality along the optical axis. Still, lenses may be designed to have a gradient-index to mimic an actual crystalline lens [108]. Fixed focal length lenticular lenses may also be used [37].

Various types of modifications may also be applied to the crystalline lens. For instance, in [79] it was mentioned a doped acrylic lens for use in measurements using ultrasound or tomography. The doping aided in visualizing its presence in optical measurements. Portions of the lens may also be made opaque or semi-opaque with paint or colorant in order to represent a cataract condition [77].

A static crystalline lens is not capable of modeling different accommodative states, even if spatially varying refractive indexes are used. In recent years, dynamic crystalline lens have started being used. These are tunable lenses that can change shape in order to mimic actual accommodation. They are typically made of a flexible material, such as a hydrogel or a silicone, that squeeze and stretch the lens to change the level of accommodation [77]. A realistic deformable lens was described in [62], being an elastomer-based refractive lens whose shape was controllably deformed using liquid crystal elastomer (LCE) actuators, allowing controlled tuning of astigmatism and other primary aberrations. The model presented in [57] used an electronically variable focal length lens made of a special polymer membrane filled with an optical fluid, having optical power proportional to the pressure in the fluid, and modulated by an electromagnetic actuator that responded to the electrical current applied. The change in the electrical current could drive the tunable lens within a range of optical power from 7D to 24D. Another electronically variable focal length lens used instead a voice coil that controlled a compression ring to compress a PDMS lens [51]. The model presented in [24] used a liquid crystal lens to control its refractive index through a computer, and the model described in [55] used a solid elastic lens (SEL) controlled by a mechanical mount designed to use tensile stress in order to change the curvature of both surfaces. In [54] a fluidic lens with a silicone membrane controlled by liquid pressure effected by piezo actuators was used.

Other stretchable lenses worth mentioning have been described in [109,110]. The former consisted of a lens with multiple refracting interfaces separating different materials, which changed its refractive properties when stretched. The latter consisted of an electrically tunable lens made of dielectric elastomers that functioned as an artificial ciliary muscle [110]. Unfortunately, both systems have yet to be implemented in an eye model.

As the crystalline lens is an internal system, it is usually attached to a holder that may or may not be detached from the device. In an interesting case, an IOL was sandwiched between two membranes, simulating the posterior capsule and anterior capsulotomy [10], despite the model was not used for optical measurements. The holder is usually a platform where a lens such as an IOL is suspended by its haptics, but some cases require the haptics to be removed in order to attach the lens [46]. In [23] it was described a groove or recess on the model enclosure where the IOL haptics were attached. More complex lenses such as dynamic ones may need specific holders, substrates [51], or circuit boards [62].

In the case of IOL performance evaluation, the holder can usually rotate and translate in various angles and directions, to change the position of the lens relatively to the optical axis, or to simulate decentration, shift or tilt of an IOL [11,12,16,22,30,32,33,36,48]. In [73,74], a fovea projector could move perpendicularly to the optical axis in order to observe different sections of an IOL, thus emulating decentration and shift. Suggested values of decentration range between 0 and 2 mm, with 0.1 mm precision, while for tilt they range between 0 and 5 degrees, with 0.01 degrees of precision. These changes in position may be performed manually or automatically, for example by using a moving device with control mechanisms controlled by position sensors to adjust the positioning of the lens [48,73,74].

An explicit crystalline lens is not often present in a physical eye model, where an IOL is mostly used in its place. Such IOL may be used either to simulate the crystalline lens [38,52], or to model an aphakic eye with an IOL implanted in order to evaluate its performance. Models with both a crystalline lens and an IOL do also exist [46,77,80], as well as models with no crystalline lens. The latter do not require enough complexity to model both the cornea and crystalline lens separately, and use a single lens to simulate both of them, as we saw earlier. In other cases, the model only requires a cornea, as described in [34].

### 5.3. Iris and Pupil

Optical aberrations, diffraction, retinal illuminance, pupil centration, and the Stiles–Crawford effect are all affected by the pupil size [19]. Therefore, an iris with a pupil is commonly included in artificial eye models, usually a light blocking surface to act as the iris, with a central circular aperture. An interesting case used a curved ring to represent the iris–lens interface, and also made it with a rough texture front surface, since the human iris is not smooth [75]. Another one used tapered edges to reduce unwanted diffraction [76].

The pupil is usually replaceable or adjustable in order to provide different diameters, typically ranging from 2.0 mm to 8.0 mm (physiological range), with increments of 1 mm or 0.5 mm. This is important for simulating different illumination conditions (photopic and scotopic) and for inducing and controlling optical aberrations.

As with the cornea and the crystalline lens, specific iris holders may be used and attached to the model, as the iris should be as thin as possible (the thickness of the iris is around 0.35–0.45 mm in humans [111,112]) and may be difficult to handle. However, pupils milled directly into the device enclosure were also found [34]. There are also cases where the holder used to attach the crystalline lens or IOL was also used as an iris [13,28], or where both parts were mounted on the same holder [73,74,76]. The holder or the pupil attached to the holder are usually replaceable.

Some eye models use instead exchangeable diaphragms [30] or a dynamic diaphragm iris [57], which may be adjusted manually or automatically [55,74]. For example, a light sensor may be used in a feedback mechanism to control the pupil size [55]. Another interesting case used a phase plate with a light limiting portion disposed around it, whose combined system made it possible to control aberration and also to function as a pupil [78].

Another interesting dynamic iris system was described in [113], which employed liquid crystal elastomers to control its aperture. Despite this device was-based in the human iris, it was not used in any eye model so far. Another relevant type of iris was used in [70], which consisted of a compact dynamic microfluidic iris for active optics previously developed by [114]. Modulation of the iris aperture size was achieved by the injection of an infrared light absorbing dye into a spiral shaped microfluidic channel. By filling the spiral with fluid, the pupil aperture would decrease.

In [62], two types of irises were presented with the purpose of accurately reproducing the human iris function. The first consisted of a liquid crystal elastomer (LCE) tunable iris, whose aperture diameter could be increased by applying an electric voltage. The disadvantages of this system were the slow time response of the LCE contraction, way longer compared to the human iris, and also the fact that it was not guaranteed that the iris would remain circular under actuation. Therefore, a second type of iris was also developed, this one being a fluidic tunable iris based on optofluidics, using a completely liquid-filled cavity and a combination of transparent and opaque fluids. Time responses for closing and opening the iris were shorter than the ones of the human iris. This second type allowed smaller aperture diameters, but only at discrete values. In addition, the optical path of the fluidic iris passed through the transparent liquid filling it and several interfaces, resulting in some absorption and reflection of light, while the LCE iris was air filled.

The pupil discussed here should not be confused with the entrance pupil, also commonly used in both in vivo and in vitro experiments. The entrance pupil is usually placed in front of the eye to limit the amount of light entering on it. As a suggestion, one could interpret the entrance pupil more as the degree of aperture of the eyelid, rather than the pupil of the eye.

### 5.4. Retina

Numerous configurations have been used to simulate the retinal surface. Here, we can distinguish between four main types: sensing retinas, diffuse reflecting retinas, transparent retinas, and active retinas. Sensing retinas always involve a sensor such as a charge-coupled device (CCD) or a complementary metal-oxide-semiconductor (CMOS). While CCDs have superior imaging qualities and lower noise than CMOSs, they also require more power and have larger pixel sizes [54]. Larger pixel sizes are more sensitive to light, allowing smaller aperture sizes, which makes it possible to decrease the amount of aberration. On the other hand, they may be less appropriate to model the size of the photoreceptors. Other sensors have also been seen, such as colorimeters, photodiodes [70], radiometers, etc. [40,70,76]. Photodiodes and radiometers are often used for radiation intensity studies and not image formation. To protect the sensor from the medium used inside the eye system, glass laminae walls are typically used. Most sensors are actually outside the device and a glass window is placed the retina plane, as happens in wet-cell models. In these cases, relay optics such as microscope objectives relay the light from the retinal plane to the sensor.

The pixel size is preferentially close to the center–center separation between adjacent photoreceptors in the human eye, which is in the range of 2.0–3.0 µm at the fovea [115]. For instance, ref. [37] used a sensor with a 2.2 µm pixel pitch, while [45] used 3.6 µm, which are both very reasonable sizes. Whenever this resolution is not enough, optical magnification can still be applied to the image prior of reaching the sensor. For example, ref. [20] had in account the ratio between the pixel linear spacing of the sensor and the cone spacing at the human fovea, and thus used a magnification relay system to correct the CCD resolving capabilities to the same level of the fovea. With respect to temporal resolution, no issues have been reported, as the frame rate of any camera is usually set up to at least surpass the temporal resolution of the retina.

On the other hand, non-sensing retinas do not use a sensor, and are typically diffuse reflecting surfaces for double-pass experiments, transparent windows for direct observation in single-pass experiments, or active surfaces with light emitting devices for second-pass experiments. The retina type directly depends on the application intended for the eye model, and combinations of different types can also be encountered, either simultaneously or through replacement of parts. For example, in [73,74], a viewing apparatus through a transparent window for live observation by a subject was used simultaneously with an external recording device; in [37] a photoactive device was replaced with a diffuse reflecting surface in the retinal plane; in [28] a transparent window was replaced with a solid retinal surface; in [57] a diffuser and a light emitting source were located at the retina plane; while in [64] two alternative types of retina modeling were used: a semi-transparent disc to allow a reflection to be obtained for wavefront measurements, and a light input from the reverse-side of the artificial eye to illuminate the semi-transparent disc, acting as an active retina for second-pass measurements.

The human retina forms a curved image plane that naturally corrects the effect of field curvature, but most sensing surfaces are planar. Planar surfaces do not allow the on-axis and off-axis beams to be in focus simultaneously. A planar photoactive area may be acceptable only whenever the interest is confined to central vision and on-axis images, for example an area close to that of the fovea. Areas up to the size of the macula lutea (3–5 mm) should be fine [116], but as the area increases, the curvature of the retina gradually becomes more significant, and Petzval field curvature aberrations start to become relevant. A major problem is the extreme difficulty of fabricating a curved sensor with such small dimensions as the ones of the retina, despite progresses in this direction have started to be done [117].

One way to circumvent the previous problem is by using a lens for field curvature compensation, to properly correct and bring into focus the image on a flat retinal plane, but this requires additional optics inside the eye model. Another solution consists in varying the depth of the posterior chamber [64] to compensate for the perpendicular distance relatively to the optical axis, when switching between on-axis and off-axis measurements. Unfortunately, this is only acceptable for punctual measurements, and does not work well when the goal is the formation of images.

More reliable solutions involve simulating a curved retina. For example, ref. [46] used a closely packed fiber-optic bundle distributed along a sensing surface that matched the curved profile of the retina (see Figure 11). The bundle was then attached to a flat surface, in such a way that the image projected onto the curved surface is mapped to a planar surface, which in turn can be detected by a conventional CMOS/CCD sensor. Each unit of the fiber-optic bundle had a core diameter of 4 µm (photoreceptor) and a center-to-center spacing of 4.2 µm (128 cycles/mm), which were comparable to the resolution of the macular area of the retina. With this approach, a retinal radius of curvature of 11.019 mm and a field-of-view (FOV) of 34.5 degrees were achieved. The small size of each fiber optic also allowed an acceptable spatial resolution for the output image using a sensor with a pixel size of 6.4 µm. The disadvantages of this method were that image processing had to be subsequently applied in order to correct the discrete contributions of each fiber and yield a continuous image, a darkening around the edges of the images caused by the limitation of the numerical aperture of the fiber, and its inability to couple light efficiently at high FOVs.

Another way of simulating a curved retina consists in allowing the sensor to move in a curved trajectory that matches the retinal curvature. For example, refs. [37,80] used a rotational arm with a nodal point coinciding with the vertex of the posterior crystalline lens surface, which could move along the retinal arcuate area. It was capable of covering up to 35 degrees of eccentricity, as well as different “kappa” angles of the visual axis. The robotic arm could also be controlled by a computer, making it possible to create and save contours and use them later to analyze a particular eye image output of a particular optics configuration. Interestingly, this approach also made it possible to change the curvature and asphericity of the retina, as the rotational stage could also translate in the direction of the optical axis, making it possible to model different physiological and pathological conditions. Other rotating retinas have been reported. For instance, ref. [30] used a recording unit mounted on a movable arm which could be rotated parallel to the retina window curvature, while [76] also made it possible to vary the position relative to the artificial eye (translation using a rod and rotation matching the curvature of the retina using a protractor) to evaluate any part of the posterior surface. However, both cases did not seem as reliable as [37,80].

The drawback of these approaches is that composite images may be required to create a specific field-of-view (FOV). In addition, care must be taken when choosing the sensor. Intuitively, one can think about acquiring an image, truncating its peripheric region (i.e., ignore all pixels outside a central region, truncating square regions of the image is a quite common practice for on-axis images, and a quick example can be found in [45]), moving the sensor to another position on the retinal curved plane, capturing a new image and using image processing to form a composite image. However, in practice, an additional problem arises, as most sensor devices are large compared with the dimensions of the eye, and cannot be easily moved widely along the entire spherical perimeter of the retina without hitting other components due to space limitations. According to [80], the area should not be greater than the foveal area or 10% of the total retinal area; however, photoactive areas between 1 and 4 mm across are generally satisfactory. For these reasons, very small sensors should be used, which eliminates many of the off-the-shelf and easy-to-mount options.

Another more realistic option to explore is the use of photodetector arrays on thin elastomeric membranes [69,118]. A recent publication described a device able to simulate the retina in a very realistic way, by using a hemispherical high-density perovskite nanowire array. An ionic liquid electrolyte was used as a front-side common contact to the nanowires and liquid-metal wires were used as back contacts to the nanowire photosensors, mimicking human nerve fibers behind the retina. The nerve fiber layer was connected to a printed circuit board. The nanowire density (500 nm of pitch) was much higher than that of photoreceptors in the human retina, and thus could achieve high image resolutions. In addition, the liquid-metal wires were located behind the sensing material, thus avoiding the light-loss and blind-spot problems of the human retina. The hemispherical shape of the sensing surface resulted in a field-of-view (FOV) with a diagonal visual field of about 100.1 degrees, with the authors claiming further improvements could cover the entire human vertical FOV of about 130 degrees.

When a transparent surface is used as the retinal surface, the focal plane is usually at its anterior surface. One publication reported focusing the image slightly behind the window in order to avoid deformations and polluting of the image formed by the optical system [30]. The image being focused on is usually captured by relay optics, such as objectives, and transferred to a sensing surface with a 1:1 ratio, or other ratios if magnification or scale corrections are desired. The rear window may also be curved, to mimic a true retinal surface. For example, ref. [77] could use either a planar window, or design it with an anterior curved protrusion, whose radius, shape and center of curvature could be controlled. The surface could also be tilted, decentered, or otherwise displaced to replicate actual eye characteristics (for these cases, alignment indicia printed on the window surface may be useful). Other publications that mention curved window surfaces include [30,75,77,79].

The main type of non-sensing retina surface is the reflecting diffuser, for double-pass and wavefront measurements, acting as a field-stop. In the case of wavefront measurements, the diffuser often makes it possible to rotate in the direction normal to the incoming light, in order to reduce speckle noise and destroy the spatial coherence of incident light (depending on the degree of coherence, the angular velocity of the diffuser, and the detection specifications) [8,9,15,24]. Other diffusing retinas have also been described: the model described in [23] used a painted surface to act as a diffuse reactor; ref. [28] used a solid painted surface to backscatter infrared radiation; ref. [79] used an end-cap made of orange acrylic; while [34] used an acrylic material for both the enclosure and retina; and [59] used cardboard of varying roughness to act as diffusers. The models described in [15,16,37,64] also used diffusing surfaces for wavefront measurements.

Diffusers have also been used for fundus visualization. Both [53,58] used an infrared reflecting surface to be imaged by confocal scanning laser ophthalmoscope. Some models used 3D-printable material to fill the entire inner surface of the enclosure, including the retinal area: ref. [52] used a photosensitive resin and [70] used polylactic acid. In these cases, markers may be useful as reference points. In [77] were used markers to locate the light spot on the retinal surface and for alignment purposes, while [20] used a graduated reticle printed on the retinal window to allow precise focusing of the relay optics on the window surface and to introduce an absolute length reference in the resulting image, used to calibrate exactly the relay magnification and to size the image itself. The interior of the posterior surface of the eye model used by [53,58] was lasered with a concentric grid with 10-degree inclinations in multiple meridians, to serve as markers in the infrared reflectance images to analyze. In [73,74], the fovea window had small letters or symbols engraved or otherwise mounted or supported on its surface, preferably the anterior surface, to help an observer subject focus on the target object. These could be illuminated, preferably when the view of the target was blocked. The model eye described in [52] had angle scale bars glued from posterior to the periphery of the retina, while the model described in [38] had USAF glued.

For second-pass experiments, active retinas are used, whether by using a point source emitter (e.g., LED), or by using retro-illumination of a scattering medium [57,64,77,80]. These photoactive retinas provide unique ways of investigating the single-pass characteristics of the front-end of the eye model, and avoid double-pass experiments, which are more error prone. Retro-illumination can also be helpful for fundus visualization.

Some artificial models have used a lens to simulate the retina instead of a diffuser [35,72]. The light would be reflected on the posterior surface of the lens, in a similar way as a diffuser. The lens used by [72] was also marked on the rear surface with concentric circles to measure the angular field-of-view (FOV). A reflection lens for double-pass experiments was used in [35]. In [76], the image plane could be transparent (plexiglass) or translucent (frosted). A frosted plate playing the role of the retina was also mentioned in [21]. The retina used in [77] could be optically or physically transmissive, diffusely or specularly reflective, or show polarizing or filtering characteristics to replicate cone function or other selective features. Its reflectance could be modified; for example, a reflectance of 10% could be used to represent a surface that is almost transparent, while a reflectance of 30% makes it possible to simulate a fundus. The reflectance of the diffusion surface could also be set to become sufficiently large with respect to the level of noise, such as reflected light from an aberration adding member.

Some devices may lack a retina representation. For example, the models described in [11,12,22] were used to evaluate the crystalline lens or IOLs using Scheimpflug or Purkinje images, and therefore did not require a posterior chamber nor a retina. Another example is [10], which used microscopy to observe laser damage to an IOL and also did not require a retina.

### 5.5. Chambers

The eye contains three internal chambers. The anterior chamber, between the cornea and the iris, the posterior chamber, between the iris and the crystalline lens, and the vitreous chamber, between the crystalline lens and the retina. The anterior and posterior chambers are interconnected and filled with the aqueous humor, while the vitreous chamber is filled with vitreous humor. The depth of the anterior chamber varies between 1.5 and 4.0 mm, averaging 3.11 mm [119]. The posterior chamber depends on illumination conditions, accommodation and intraocular pressure, due to degree of aperture of the pupil and curvature of the crystalline lens, with values between 0.06 and 0.26 mm, with uncertainties around 0.10–0.15 mm having been reported [120]. The vitreous chamber depth varies from 14.42 mm to 16 mm [121]. Reported lengths of the three chambers in artificial eye models are mostly inside the physiological range.

Anterior segment chambers’ depths are typically reported as distances between the cornea and pupil, and pupil and crystalline lens or IOL. Due to its small length, the posterior chamber is often neglected and the crystalline lens is placed immediately after the pupil. However, accurate depths have also been modeled, for example, ref. [37] used an accurate value of 0.2 mm plus an additional contribution given as a function of accommodation. In [45] posterior chamber depth of 0.45 mm was used.

The distance between the cornea, pupil and crystalline lens is typically constant, but some models make it possible to vary it. One way of doing so is by shifting the crystalline lens/IOL along the optical axis direction. Many eye models allow shifting, decentering, and tilting the lens, as seen before, and this usually includes shifts in the optical axis direction. Wet-cells can simply change the thickness of the cuvette, or change the relative positions between optical components on the optical bench. Some other models have specific mechanisms to vary the distance between these components, but they usually require disassembling the device to apply the modifications [39,46,52,73,74,76], or building alternative embodiments of the model [12,52].

On the other hand, the length of the vitreous chamber is commonly variable, making it possible to change the distance between the retina and the nodal point of the eye (typically the intersection between the optical axis with the posterior surface of the crystalline lens). By moving the retina relative to the crystalline lens (or vice versa) in the optical axis direction, it is possible to change the total refractive power of the eye model. Typical lengths used for the posterior chamber also fit inside the physiological range. In wet-cell models, one simply needs to change the relative distance between the wet-cell and the camera on the optical bench, and adjust the respective relay optics. For example, ref. [25] mentions a motorized translation stage to move the microscope along the optical axis. In [13], the distance between the IOL and the artificial retina was variable and could be adjusted by means of a micrometer screw. The retinal plane was fixed while the lens plane with the anterior chamber could be moved forward and backward. In [33], it an imaging system including the CCD was described that could be moved along the optical axis for different focus positions behind the IOL. In [30], the camera with the microscope objective could be moved along the optical axis with steps of 0.01–0.05 mm.

More realistic models less dependent on optical benches and with an integrated retinal surface need more elaborate ways to change the axial length. For instance, ref. [46] described all of the optical components, including the artificial retina, being mounted into the housing so that the spacing between any two elements might be adjusted to match the anatomy of individual patients’ eyes. In [37,80], the sensor was mounted on a motorized translation stage with linear movement up to 6 mm, which allowed simulation of ametropia, from approximately +3.00D to −16.00D. By combining rotation and translation, the radius of curvature and asphericity of the virtual retina could be controlled. In [28], a threaded sleeve allowed the length of the test eye to vary until the desired refractive error was observed. In [20], the overall axial length (distance from corneal apex to the retina) could be varied micrometrically between 20 mm and 30 mm, allowing it to host high-power IOLs as well as to simulate an aphakic eye, while the camera was mounted on a micrometric translation stage. In [24] it was used a retinal plane, mounted on a movable platform manually controlled by a micrometric screw. The invention described in patents [73,74] used a fovea projector that could protrude into the eye model, thus varying its axial length. Patent [75] stated that the relative position of the CCD camera and the lens system could be varied while patent [76] claimed that the artificial eye’s refractive state could be changed by adjusting the distance between the lens holder and the interior surface of the posterior portion. In invention [77], the separation distance between the retinal surface and the posterior corneal surface was made variable via flexible housing joints. The device used by [53,58] also appeared to have configurable distances, as both authors reported different lengths in spite of having used the same device. In [45], the artificial vitreous chamber could be moved by means of a motorized translation stage, providing an accuracy of 2.5 µm.

Ideally, the anatomic size of a physical eye model should be in the range of real eyes, with possibilities of modifications to account for age, disease, ethnicity, or other natural variations. Therefore, the total axial length of the eye should range between 15 and 30 mm. While most eyes follow these specifications, there are some exceptions. The eye model created by [42] was actually 1.336 times smaller in relation to that in the Gullstrand model, to compensate for the removal of the liquid used to fill the model as the CCD camera used was not waterproof. The shrinkage factor was equivalent to the assumed refractive index of the fluid, i.e., 1.336. On the other hand, the model proposed by [76] was about 6.5 times the size of a natural eye, perhaps to make the mounting process less difficult and to facilitate high intensity radiation measurements. Nevertheless, when the goal is to provide reliable measurements, such as image formation, the size of the model should be as accurate as possible.

### 5.6. Aqueous and Vitreous Fluids

Most eye models are at least partially filled with a medium to simulate the eye humors. The anterior segment chambers of the eye are filled by the aqueous humor, while the vitreous chamber is filled with vitreous humor. The vitreous chamber is a separated cavity of the eye and the aqueous humor and the vitreous humor are fully separated and distinguishable fluids. The aqueous humor is similar to a water solution, while the vitreous humor better resembles a gel. While there is a preset amount of vitreous humor in our eyes, and it does not move freely, the aqueous humor is continuously produced and recycled and flows through both the anterior and posterior chambers.

Most artificial eye models do not distinguish between the two fluids, and for simplicity, their internal cavities are modeled as single chambers or wet-cells, with a single fluid. There are indeed rare situations where the chambers are fully or semi-separated [13,76,80] and at least one case in which different fluids have been used [76]. The model presented in [64] also seems to have separate chambers. Curiously, refs. [13,80] allowed both chambers to communicate through shunts or gaps.

The simplest mediums for filling the internal chambers of an eye model are water or distilled water [10,11,13,45,46,75], purified water [32], or deionized purified water (n = 1.334) [37], but more representative fluids can also be used, such as saline solution [33], balanced (phosphate) saline solution (BSS, n = 1.334) [20,38,52,70], physiological solutions [19], or 2.1% sucrose water solutions (n = 1.336) [28,48]. Sucrose water solution is relatively popular, because its dispersion is well known and has been widely published [122,123]. Other more complex solutions have been reported, such as polyethylene glycol diluted in water [27], or fused silica matching fluids [64]. The latter was chosen in order to achieve almost the same refractive index of the fused silica cornea used in the same model, and avoid unwanted reflections. For the vitreous fluid, gel-based solutions may be a good choice, such as gelatin mixed with water [76], transparent fluid gel [77], but usually the same fluid used to model the aqueous humor is also used to model the vitreous humor.

When designing a possible solution, the Sellmeier coefficients may be helpful, as they contain all information about dispersion. Factors to consider when preparing a medium, are the temperature, which influences the refractive index, and its chemical components, which may affect dispersion relations and rotations of planes of polarization. In [48] was suggested a mix of sucrose and sodium chloride to avoid these problems, despite not having used it. Since the medium might act as an electrolyte, the components of the eye system have to be chosen carefully by taking into account their electrochemical properties, especially if they are metallic [48]. Chemical reactions may contaminate the medium, changing its optical properties, and erode and damage components of the device.

One disadvantage of filling with fluid artificial eye models with sensing retinas is that, unlike the natural retina, which is in contact with the vitreous fluid, the same cannot happen with electronic sensors, which require a glass protection separating the sensing surface and the liquid. Wet-cell models use the walls of the wet-cell, but other models have the retinal surface in direct contact with the aqueous fluid, which is usually water-based, and unlike a gelatin, can easily flow to the interior of the sensor and damage the light reactive surface. Commercial CCD and CMOS sensors already have a front-end glass protection (which can be waterproof or not). This means there is always a small air space between the eye model and the sensing surface that cannot be removed. It is usually a challenging task to properly separate the chamber medium from the sensor without affecting the light propagation characteristics. This is usually solved by the use of transparent retinas and relay optics to properly focus the light from the retinal surface the sensor’s surface with a 1:1 magnification ratio, or other magnification ratios such that each pixel of the sensor matches approximately each cone. However, this usually requires increasing the distance between the glass wall and the sensor to give enough space for the relay optics and their respective focal lengths. This is also the reason most wet-cell models have their sensor some distance away from the glass wall.

More drastic solutions involve removing the whole fluid from the system and use an air-filled eye model. While air-filled eyes can be acceptable for certain types of measurements, such as in [53,58], the liquid’s removal violates the asymmetry in the object and the image space resulting in an incorrect image magnification and a different range of the field depth [42]. For this reason, some scaling corrections or lens properties adjustments may be necessary.

Some devices make it possible to pressurize the medium, in order to simulate intraocular pressure, which can be useful when testing immersed IOLs. Refs. [37,80] controlled the volume and pressure of the fluid using a piston pump and the elastic properties of the bladder-like chamber. In [73,74], the entire device (except the external part of the fovea projector) was made a single air-tight compartment, making it possible to pressurize the eye model. PAtent [77] also made it possible to pressurize the fluid by using a valve system at the inlet/outlet.

Finally, in order to avoid contact lens evaporation, humidity/temperature chambers closed by transparent glass have also been reported [37,45,77,80].

## 6. Housing and Assembly

To make an eye model functional, it is necessary to properly assemble and align all of its components. Most physical eye models are mounted on an optic bench, but the way their individual parts are assembled together varies between devices.

Simpler models such as wet-cell-based models are not difficult to assemble. An IOL is usually immersed inside a cuvette which is then mounted on an optical bench and aligned horizontally with the remaining components (see Figure 3 and Figure 4). Different components are usually not attached to each other, but instead to the optical bench. These models have little to no housing. Moving optical components along the optical axis is relatively simple. The most difficult part is perhaps the task to properly align the IOL inside the cuvette, and to apply diverse degrees of tilt, decentration and translation in a controlled and precise manner. These setups are typically used to test IOLs, and could be seen more as an in vitro bench setup rather than an artificial eye model. While it is very simple to mount these physical models, they have the disadvantage of requiring an optical table, and thus can only be used in laboratorial environments in most cases. They also need some time to mount on the optical bench and prepare each experiment. On the other hand, they can be highly configurable and be set up to fit different specific needs, by simply moving or replacing parts on the optic bench. Examples of wet-cell devices mounted on an optical bench are [13,14,18,19,25,27,29,31,33,43,47,49,50,56,60,65,68,71].

To better resemble the anatomy of the eye, some physical models are packed into a single unit or closed system, with all their parts attached to a single housing enclosure, as seen in Figure 5, Figure 15 and Figure 19. These devices are typically very small, compact, robust, portable, and ready to use. Their enclosure is usually spherical or cylindrical to mimic the shape of the eye, but this is often done only for aesthetic purposes. Other shapes also exist (see Figure 6 for an example). They can also be mounted on an optical bench, or attached to other hardware, which gives them some versatility. Usually, these devices comprise of two halves that are attached together for easier mounting, but some devices may include more or less parts. Some configurability may exist, allowing simple modifications such as variations of the axial length of the system using threaded sleeves, but unfortunately, these models usually have fewer features and are often less configurable than more complex devices, can be hard to fabricate and assemble due to their small size, and in some cases the parts are glued or cemented together, meaning they cannot be disassembled, adjusted or replaced after the initial assembly [46]. Despite being esthetically more attractive, these devices may also lack some configurability. Some examples can be found in [23,34,35,38,40,42,46,51,52,53,58,62,63,70,76,79].

The most interesting and complex devices are a hybrid version between a single unit device and the optical bench setup. These devices are the most creative and experimental, and therefore there is a significant variety of them, while they are also often highly configurable. They are usually large and bulky, in order to accommodate all the structure needed to provide high configurability and complexity, while their interior is typically a closed system such as compact devices. The use of micro-bench mounts is a common characteristic, and they may also have several joints, sliding parts, rods, threaded sleeves, micrometer screws, robotic arms, viewing apparatuses, fluidic elements, different types of sensors, and electronic components for computerized control. Relative movement of different parts is usually precisely controlled for proper alignment. Despite being larger than packed units, it is not difficult to move these devices and install them on optical benches, and while they are not aesthetically attractive, seeming to have a bulky and dirty mounting, they are much better in reproducing the eye behavior. The main drawback is that they are often hard to design and mount, and are not very intuitive to use for someone not familiar with them. Some examples are [30,32,37,45,48,54,55,57,64,73,74,80]. Figure 16 shows a typical architecture of this type of devices.

The use of holders or carriers to contain optical components is quite common in eye models. One simply attaches the component inside the holder, and then the holder is introduced into the eye device enclosure. While it is possible to attach the optical parts directly into the enclosure (using drilled boreholes [12], inserts [46], grooves, recesses, etc.), their small and thin sizes make it much harder to handle compared to a carrier, which is much easier to move and also offers protection to the sensitive components. This is particularly useful when replacing parts, as one can simply replace the holder without needing to touch the sensitive components, or just remove the holder and replace the parts in a more controlled environment. Another utility is to provide a universal holder to alternative optical components from different sizes and/or manufacturers, which may not be easily attached to the same joint (e.g., IOL diameter, shape, or angle of haptics). Therefore, the use of holders with only subtle modifications is an easy way of achieving compatibility. If a new component needs to be used, a new holder may be easily manufactured through additive manufacturing techniques such as 3D printing, or other machining techniques. One should not forget that the material should be inert in the presence of the filling medium.

Holders may also be used to align and adjust the position of lenses through precise control mechanisms without needing to disassemble the device. This is often done to facilitate controlled IOL shifting, tilting, and decentering. These specific holders are sometimes referred to as micrometric stages, translation stages, rotational stages, XYZ-stages, three-axis stages, etc. [11,22,29,45], which can be controlled manually (through screw gauges for example) or automatically (through robotic arms for example). The only disadvantage of using holders is the extra space they occupy, and the requirement for custom machining.

In addition, holders can also manage multiple optical components, and make it possible to vary the relative position between them, making it much easier to alternate between embodiments of the eye model. As an example, we consider the eye model described in [76], where a single holder was used as the entire anterior segment and could be mounted completely independently from the remaining of the device. This included a thread that made it possible to change its depth, and its interior was filled with aqueous fluid. This would then be inserted into the front of the main enclosure of the device comprising of the posterior segment, which was filled with a different vitreous fluid. This way, both humors would not be in contact with each other.

The housing of an eye model is typically a metallic, acrylic, resin, or other hard polymer enclosure, creating a closed structure around one or multiple chambers. Some cases started with a non-hollow housing and a hole was drilled to form the interior chambers [46]. The housing should be opaque enough or at least its internal surface should be blackened to reduce stray light. The housing may be split into various pieces that are assembled together to close the system. Different parts of the housing and components may be attached with screws, locknuts, etc. The housing usually has specific slots, joints, grooves or recesses, where holders and components are attached when mounting the system. In [46], a clamp was also used to hold a curved fiber optics bundle.

The material chosen for the housing must be approximately inert in the presence of the internal liquid. Inlets and outlets may be used to fill the internal chamber, while in other cases the chambers must be filled before sealing the system. Metal spacers may be used to separate individual components, and rubber seals or o-rings are often employed to prevent fluid leakage. Flexible enclosures have also been used [37,73,74,80] in order to avoid fluid leakage when the axial length and volume of the chambers change, but other solutions might be possible, such as using elastic tubes attached to the chambers’ inlets.

When mounting or replacing a component in the eye model, it may be necessary to (re)calibrate the position of all components of the device, to ensure proper alignment with the optical axis and avoid unwanted aberrations such as astigmatism or coma. Holders make it possible to minimize this problem, but are not guaranteed to eliminate it completely. Some devices use high-precision movable holders or sliding parts of the model housing to meet this goal. For example, ref. [35] achieved precise alignment through the use of two different micro-bench mounts linked by rods, and screws allowed precise sliding movement between them. Microscopes may also be used to help aligning and spacing different components [32,46], while other optical centering instruments have also been reported [54]. Another way is through the use of tracking cameras placed in front of the device to provide information about the fit, centration, or meridional orientation of components [37,63].

A common challenge when mounting a physical eye is how to deal with the internal fluid that is used to model the aqueous and vitreous humors. Different ways have been employed to fill the artificial eye chambers with liquid, and it depends a lot in how the device is mounted. How the chambers are filled is not always reported, but there are some known examples. For instance, the fluid may be inserted/removed through inlets and outlets [12,20,28,30,46,76,77]. In other cases, the fluid is inserted during mounting of the device, before sealing it. In [73,74], the liquid was filled by the cornea aperture, which was later sealed by the cornea lens. In [76], the anterior chamber consisted of a lens holder which contained the cornea, pupil and crystalline lens. To fill its interior with fluid, it was necessary to remove one of its end-parts. Invention [75] used an intraocular assembly, with at least a front-end lens and a rear-end lens, creating a chamber capable of holding liquid, using water drums. To prevent fluid leakage, o-rings may be used between adjacent parts of the artificial eye [20,46,76]. Some devices may also use an elastic bladder-like chamber instead of a rigid compartment as the posterior segment of the artificial eye [37,73,74,80]. A soft tissue wall is suitable for covering any movable joint, and is flexible enough to keep the chamber sealed as the posterior wall changes position without interfering with the retinal image projection. In [73,74], a shaft seal was alternatively used. The device presented in [64] was designed in such a way that the total axial length and volume of the enclosure was kept constant when moving the IOL between the cornea and retina, facilitating the sealing of the system and avoiding leakage. Another and more extreme way to prevent leakage is by gluing or cementing the components [46,78,79]. However, this approach does not make it possible to easily disassemble the eye model in order to replace components.

An additional problem of liquid-filled devices is the presence of air bubbles, which may be present in front of the optics and/or sensing surface, affecting the image quality. Some devices employ methods to minimize this problem. For example, a syringe pump may be used to fill the chambers, while helping to avoid the formation of air bubbles [20,30]. Another method is to use vacuum to remove air bubbles [46]. Other approaches involve mounting the device vertically instead of horizontally: Invention [79] was not able to remove air bubbles, but instead included a flange to handle the device and prevent air bubbles of migrating to the area above the cornea. Air bubbles were also forced to migrate by gravity across the crystalline lens region to the retinal area where the flange was located. The device could be centrifuged so that air bubbles would enter the flange. In order for this to work, the model had to be in a vertical position when used (the device was used upside down, with the retina on top). A feature of the model described in [80] is the provision of gaps between the iris ring and the lens to permit air trapped in the posterior chamber to gravitate upward into the anterior chamber. The device presented in [45] was also assembled upside down, while the vitreous chamber was closely attached to the anterior eye segment. After water filling was finished, the model eye was attached to the translation stage in correct orientation, and the vitreous chamber was separated from the anterior segment, so that their relative distances could be varied.

Another reason we may want to mount an eye model vertically is to take advantage of gravity to avoid the internal fluid leakage. Other devices were mounted vertically for practical purposes only [37,80]. Some devices for fundus viewing may also be placed in a vertical position, so that an observer may look into them with the device resting on a table, as if he was looking at a microscope [38,52]. The disadvantage of vertical systems is that they may require folding mirrors in order to receive input and direct output to instruments, which are typically positioned horizontally in the laboratory or optical bench.

## 7. Eye Movements

The main body of this review is concluded with a brief reference to the simulation of eye movements using artificial devices. While this is not very relevant for most eye model applications in optometry, it may be significant for certain human vision applications, and also when we want to make a bridge between computer vision and human vision. 

In the absence of binocular vision, eye movements may be used for scene reconstruction, depth analysis, object detection and tracking, or texture analyses. Evaluating how the human eye contributes to these tasks might have applications in improving vision under diverse conditions, such as night driving situations. This may also be important for industrial applications, in order to mimic how a human would evaluate an object in different angles and distances, and take appropriate decisions accordingly to what he observes.

While eye model devices with capability of evaluating binocular characteristics of vision have previously been discussed [59,74], and moving a target relative to an optical system is quite easy, artificial oculomotor rotational movements have rarely been mentioned with respect to physical eye models. A notable case is [70], where the eye made it possible to rotate relative to a light source, by rotating a platform, on which the model eye was placed, relative to another platform with a light emitter, so that the light could hit a retinal sensor at different incident angles. The first platform would slide under the second platform, to avoid collisions between both platforms. Unfortunately, this feature was only used to evaluate a decrease in light intensity hitting the retina with increasing incident angle, and not to form an image of a target viewed under different angles.

A more interesting case made it possible to simulate saccadic movements by changing the focus of an image, but without actually moving the eye [54]. In this work, composite images were obtained in order to obtain a single continuous scene, to mimic how the brain compensates the loss of image resolution with eccentricity, due to the decrease of cone density as we move to peripheral retinal areas. Aberrations such as coma may also be compensated by these mechanisms. In the real eye, the composite image is formed by a sequence of processed images acquired through saccades, quick eye movements that redirect the center of gaze from one point to another. By using the eye device (as already described previously), this behavior was modeled by using a combination of an aspherical hybrid diffractive-refractive lens with a flexible fluidic membrane lens, allowing the implementation of a light sensitive and wide-aperture optical system with variable focus. It is also intuitive that the same model could be used to model other types of eye movements, such as smooth movements to track objects, or micro-saccades to stabilize gaze. Both situations could help us understanding how fixational eye movements impact the image quality on the retina, and might also be extremely useful in an industrial context.

A final interesting example of artificial eye movements is a publication describing two robot prosthetic eyes and its control and sensing mechanisms [89]. The prosthetic eyes could move horizontally in synchronization with the movement of the natural eye. One of the devices used an external infrared sensor array mounted on the frame of a pair of eyeglasses to detect the natural eye movement and to feed the control system to drive the artificial eye to move with the natural eye. A second device instead used the human electrooculogram (EOG) signal picked up by electrodes placed on the sides of a person’s temple to carry out the same eye movement detection and control tasks.

## 8. Discussion

The human eye is a very complex system, which has a large set of characteristics that can be modeled, such as the shape of the cornea, aperture of the pupil, variations in shape of the crystalline lens, the presence of tears, axial length, the aqueous and vitreous humors, dispersion along the optical path, the retina curvature, photoreceptor density, retinal diffusion, and the presence of ophthalmic systems, such as spectacles, CLs or IOLs. All of these in combination induce various types of effects that define our vision quality. Despite its good optical performance, the eye is not a perfect optical system, being limited by physical phenomena such as lower and higher order aberrations for increasing apertures, diffraction for smaller apertures and scattering due transparency losses related with the biological structure of the tissues, pathological conditions or aging. 

A physical artificial eye model is a device whose goal is to mimic in vitro the human eye’s anatomy, behavior, or both. An ideal eye model would be able to simulate all main physiological conditions of the human eye, being capable of producing wide-field images, while also being able to be characterized using ophthalmic measurement equipment. Ideally, a sensing retina used to acquire images should be easily replaceable by a diffusing surface used for double-pass, second-pass, wavefront measurements, or fundus imaging. With information such as the MFT and Zernike coefficients, a full characterization of the artificial eye and its expected image quality under various conditions could then be achieved.

While it is desirable to have a complete eye model capable of simulating all the characteristics of the eye, this degree of complexity is very difficult to achieve. It is preferable instead to keep the model as simple as possible, and model only the few particular features necessary for its desired application. While this may sacrifice generalization, it also simplifies the construction and can make the artificial eye more reliable. Additionally, an artificial eye device does not need to reproduce perfectly all the anatomic characteristics of the natural eye, being enough to reproduce its functional behavior in most cases. The final choice of the optical design should be a compromise between the simplicity of the design and its ability to imitate the desired characteristics of the human eye. For example, if higher-order aberrations are not intended to be investigated, lens design can be greatly simplified.

Due to the high number of applications, it is remarkable that, while there are many devices htat are similar or based on others, we still have at our disposal a huge variety of different techniques and methods for building, testing and using artificial eye devices. Therefore, the current state-of-the-art technology available allows us to build and use an artificial eye for almost any purpose and way of operation. Several of these devices were discussed in this review.

The human eye is an aberrated system. Along with other sources of image quality degradation, lower- and higher-order optical aberrations affect the visual performance and are usually addressed by prescribing optical appliances in the form of spectacles or contact lenses (CLs) as well as surgical procedures to reshape the cornea or implant artificial intraocular lenses (IOLs). It is then not a surprise that aberrations are the most intensively studied characteristic of the eye, and this is not an exception when eye models are used. Low-order aberrations correspond to piston (zero-order), tilt or distortion (first-order), defocus and astigmatism (second-order) aberrations. Higher-order aberrations correspond to coma and trefoil (third-order), aspheric aberration (fourth-order), and so on. Aberrations of zero- and first-order do not affect image quality and are rarely studied in ophthalmology. Other higher-order aberrations are almost insignificant.

We can also categorize optical aberrations into axial (longitudinal, central, foveal, on-axis) and field (off-axis). Axial aberrations occur in the field center and are the ones with the greatest impact in visual quality, as they affect mostly the foveal vision. Off-axis aberrations vary with eccentricity. Because the eye operates in an inherently misaligned mode, with surfaces lacking rotational symmetry to a significant degree, typical off-axis aberrations may also occur at the fovea. One important consequence of this is that, as we increase the diameter of the pupil, all eye aberrations are amplified with the impact on foveal image quality.

Second-order aberrations account for roughly 85–90% of the total aberration of the average eye, but are also the easiest to correct. Defocus takes the biggest slice in terms of magnitude, and corresponds to well-known cases of myopia and hyperopia. Therefore, most eye models include features that make it possible to change their axial length. Off-axis defocus is often associated with the field curvature of the retina and is also widely modeled. Astigmatism often originates at the cornea, and is prone to relatively weak cylindrical deformations. Off-axis astigmatism is the most significant field aberration, but is unimportant for eye acuity in everyday life, with possible exceptions in night scenarios.

Higher-order aberrations are much smaller than defocus and astigmatism. However, they are much harder to correct, or are even uncorrectable, and may become the limiting factor for the eye’s imaging quality once the low-order aberrations are corrected. There are coma results regarding the inherent misalignment of both corneas and eye lenses that are tilted relative to the visual axis, with the lens also being somewhat tilted with respect to the cornea, and decentered. Coma in general is insignificant compared to defocus and astigmatism, and is often compensated by saccadic movements. Trefoil is rarely mentioned in ophthalmic studies, and its magnitude is typically close to that of a coma. It is affected by the cornea shape and is minimized by the aspheric (prolate) shape of its optical surfaces. Spherical aberration is generally harder to correct, with a higher impact on the average population. For this reason, it is a hot topic in most studies involving eye models.

Among the publications included in this review, defocus and spherical aberration are the optical aberrations that have been most widely discussed in physical eye model studies, as it is relatively easy to change the axial length of eye models, and spherical/aspherical lens design is a technology that has been widely developed. Astigmatism has also been often studied, but not as much as the previously mentioned two aberrations, and has typically been associated with studies involving dynamic crystalline lens. Coma has not been commonly included, appearing generally in studies comparing multiple aberrations, or studies involving tilt and decentration of optical components, while trefoil has rarely been mentioned.

None of the previous aberrations have been dependent on wavelength or are monochromatic. On the other hand, chromatic aberrations occur when different wavelengths are focused at different points. Here, we distinguish between longitudinal chromatic aberrations (on-axis) and transverse chromatic aberrations (off-axis). The latter, like other typical off-axis aberrations, is commonly present in the eye on-axis as well. Chromatic aberrations are not so apparent in vision quality and depend on many factors, such as neural processing and the cone distribution for each wavelength. Nevertheless, there are several studies involving eye models focusing on chromatic aberrations [26,37,40,52,63,64], and one theoretical eye model (the “Indiana” eye model) [124] is also a popular reference.

Despite several devices having been developed, they are not easily accessible for testing, reproduction, and comparison. This is a bigger concern in publications where the artificial eye is only a secondary tool for the purpose of the research conducted. In general, each one of the devices reviewed in this publication seems to have fulfilled its intended application up to a certain extent, accordingly to what has been reported by the authors. This makes it difficult to decide which of them performs the best for general purposes. It should also be noted that most authors tend to have an optimistic view when discussing their own research findings, and thus the reported performance of most devices presented here might be on the optimal end of the spectrum. Table 4 presents a brief comparison of all eye models included in this review that can be used to create a retinal image, with emphasis on their main strengths and weaknesses. Basic characteristics such as replaceable parts or variable power are a must-have, and will not be considered strengths. All cases, whenever the crystalline lens is not modeled by a mere IOL, are considered as strengths and called phakic eye models. Whenever off-axis vision is present, it is also marked as a strength. On the other hand, whenever relay optics are needed between the model and the target or sensor, it is marked as a weakness. Models based on wet-cells are also considered a limitation due to their extreme simplicity. As for the remaining types of eye models not included in Table 4, the obvious limitation is that they cannot acquire retinal images.

Designing and building an artificial eye with capabilities similar to those of humans is a demanding task, due to the high number of parameters affecting the final device characteristics. Perhaps the greatest challenge is its small size relatively to its big complexity, and the fact that extreme accurate optical systems usually have small tolerances. To help overcome most of the problems that one may encounter, some guidelines that may be useful for a physical eye model project are provided in the next paragraphs.

The first thing to consider is which anatomic features and functional behavior we wish to model. This will determine the complexity we will need to add to the device, which in turn defines the optical components included, its geometry, and the materials used. It may be useful to create a schematic model for theoretical simulations prior to start constructing a physical device. A schematic model is useful to help deciding some of the features of the future physical device we want to include, while can also be later used to validate it, by providing a range of expected results of future experiments. Optical modeling software such as ZEMAX, or other physical modeling software such as COMSOL Multiphysics, are good options to create schematic eye models. Checking existent models is also a good starting point. One of the purposes of this review was to provide a database of such models. To help choosing the most appropriate geometry, optical parameters, and materials, it may also be useful to look at existent schematic eye models. Popular publications about schematic eyes can be found in [1,23,92,93,96,124,125,126,127,128,129,130]. It should be stressed, however, that even schematic models are not accurate enough to perfectly model the human eye and one should never assume their output as being the ground truth. 

To match the focal length and other characteristics of the human eye, materials with proper refractive indexes have to be chosen. Therefore, the shapes and optical properties of the components must be in accordance, and each component may influence the choice of the remaining components. As an example, ref. [64] used a fused silica refractive-index-matching fluid in contact with a fused silica convex-planar cornea, in order to mask the effects of using an inaccurate planar posterior cornea suface. This shows the disadvantage of using inaccurate lenses to model physiological lenses, as by introducing restrictions on the materials used for the medium, other eye characteristics such as dispersion may be sacrificed in terms of accuracy.

Spherical surface lens models can only match the first-order properties of the eye and do a poor job in matching aberration content or off-axis properties of the real eye [17]. Aspheric eye models are much better suited to illustrating clinical levels of longitudinal aberrations, both on- and off-axis. Such models need a proper combination of characteristics between the cornea, crystalline lens, pupil size, dispersion, or medium refractive index, to match a fit to a specific aberration (spherical, chromatic, etc.). One such model is the well-known “Arizona” eye model [26], which can be used as a good starting reference.

**Figure 21:**
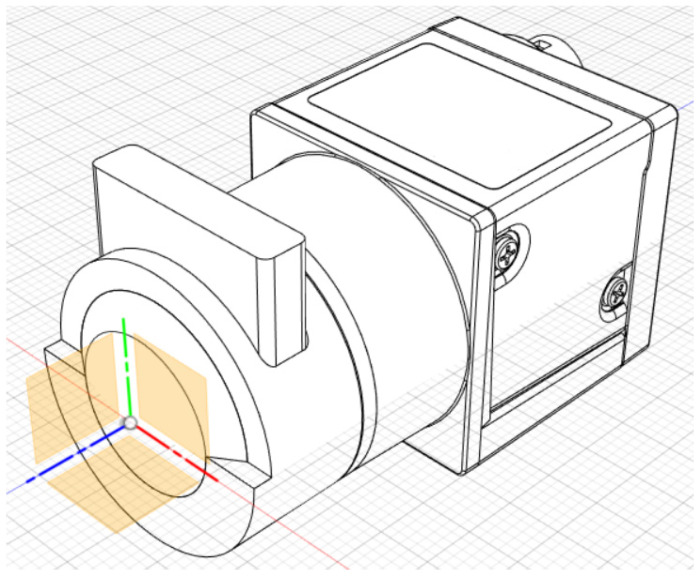
3D design of an eye model prototype using the software Autodesk Fusion 360© 2020 Autodesk, Inc. (version 2.0.12670, student license, sourced in Lisbon, Portugal). The axes’ origins are placed at the center of the anterior surface of the cornea, coinciding with the optical axis.

When designing the eye model, it may be useful to define a coordinate system. The positions of all components may then be referred in terms of the origin of that system of coordinates. This is extremely useful when designing the device parts using 3D modeling software, and also when control software is employed to adjust the dimensions of the device, or translate, shift, tilt and rotate components. According to Gullstrang, the corneal axis is the eye’s most convenient reference axis [1]. Figure 21 presents the 3D model of an eye model prototype designed using the 3D modeling software Autodesk Fusion 360, where the axes origin is at the center of the anterior surface of the cornea, coinciding with the optical axis.

Another important consideration when planning a physical eye model is its integration with measurement instruments. Single-pass measurements may only require a camera or a photodiode, while devices for double-pass measurements or fundus imaging may be better connected to wavefront sensors, or ophthalmic instruments. How this interface is done is a crucial requirement of the device planification.

**Figure 22:**
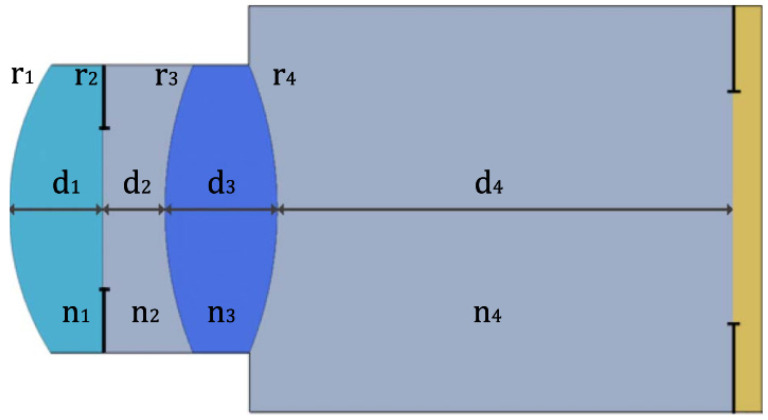
Schematic layout of the eye model presented by Coughlan (2017), where symbols denote the properties of the different components of the model. A separate table matches each symbol with the respective value. Applied Optics. 2017, 56, 4338–4346 ([64]). Originally published by and reprinted with permission from Optica Publishing Group.

A common problem found when writing this review was the lack of information about the dimensions, distances, and optical properties of some components in published eye models. In most cases, this information was missing, because it was not relevant for the intended research study, and was thus omitted. Nevertheless, this voids any possibility of reproducing the exact same device that was created in the publication. It is, therefore, suggested to include a comprehensive diagram such as the one in Figure 22 [64], reporting through symbols the properties of the different components of the model. A separate table may then associate the symbols with the respective values and units. Arrows may be used to denote variable dimensions, translations, rotations, etc. By knowing all of the properties and dimensions of the model, it is possible to create an equivalent model to the one reported, even if the construction and assembly methods are not disclosed.

It is important to account for potential sources of error during the mounting and assembly of an artificial eye model, and properly report them. Publication [48] offers some good insight into how to deal with the mounting process in practical terms, listing some of the most common difficulties and sources of error that might arise. Perhaps the most relevant one is related to the misalignment of components when assembling the device, and replacing or moving parts. It is important to make sure such movement is steady enough, and to calculate and correct or recalibrate any systematic misalignment. Using a precision tracking camera to monitor the exact positions of the components is a good tool for helping to correct position errors. The choice of the motor driving mode may also be relevant in order to avoid resonant frequencies of oscillating parts such as attached IOLs, which may introduce additional uncertainty regarding positioning [48].

Other important sources of error are manufacturing tolerances and limitations. Unlike theoretical models, the shape and dimensions of a physical model are not exactly the planned ones, and there is always a certain degree of uncertainty due to manufacturing tolerances. There is also the uncertainty introduced by the measurement equipment. Unfortunately, none of these sources of uncertainty are commonly reported in most publications.

Monitoring the working specifications of the CCD/CMOS sensor used is extremely important when evaluating vision performance. In [20], a performance “paradox” for different pupil sizes was reported: when the pupil size increased from 3.0 mm to 5.0 mm, one would expect a decrease in image quality due to increased spherical aberration. However, with the increased pupil size, the amount of light irradiance reaching the sensor led to a better signal-to-noise ratio, which was actually able to increase image performance. This problem may be difficult to detect especially when the sensor applies automatic dynamic range adjustments. Other sensor characteristics such as sensitivity, nonlinearity and noise should also be considered. The saturation threshold of the CCD camera needs to be avoided, which can limit the light intensity used in some experiments with artificial eyes, that would otherwise be used in real eyes, especially when performing psychophysical threshold experiments.

An underestimated source of error is the “kappa” deviation. Most published physical eye models do not consider that the optical axis does not cross exactly the center of the pupil, and mount their components perfectly centered around the optical axis. A real eye has the pupil typically shifted 0.3 mm nasal to the optical axis. This decentration introduces additional aberrations such as coma. Temperature is also often underestimated during ophthalmic studies, but can as greatly influence aberrations, as the refractive index is dependent on temperature. This is more significant with fluids, which have larger temperature coefficients, and can thus affect dispersion. This may be a source of discrepancies between theoretical and experimental results. A good recommendation is to use a temperature around the ambient range 20–25°C, and make sure values used for theoretical simulations match the experiments.

In human vision applications, the goal is to quantify the image quality as a human observer would do, simply by “seeing”, i.e., visualizing the image as it would appear on the retina. In other words, to analyze optical images unprocessed by the brain. The most common way to do this is through psychophysical tests. Therefore, a good eye model for human vision should be capable of reproducing the results of psychophysical tests. An impediment for this is the neural component, such as brain adaptation or eye dominance, which typically influence the results of these tests. Physical eye models only mimic the optical performance, and the actual psychophysical effects contributing to the visual performance would have to be included only via assessing the neural transfer function [131].

The work described in [20] tried to address this problem, by reintroducing the brain’s skill to identify patterns and shapes. This was done by asking a subject to recognize and read optotype characters of variable size and contrast, “seen” by the model eye and displayed on a television monitor, or analogously to guess the orientation of sinusoidal patterns of variable spatial frequency and contrast. Since the image on the monitor was magnified (presumably until the observer was satisfied), the subjective visual performance of the reader was not involved in the procedure, resulting in objective, quantitative data from simulated psychophysical tests. By varying the optical conditions of the physical eye model (e.g., used IOL), but keeping identical testing conditions, the data obtained would be directly related to the optical and visual performance of the device to those optical conditions. In [66], psychophysical thresholding tests were performed to obtain quantitative information related to halos produced in response to a glare source, which could then be compared to numeric data obtained from the eye model. While it did not account for neuro adaptation effects, the work showed that subjective human tests can be quantitatively compared with objective simulated measurements.

Physical eye models make it possible to simulate the human eye in vitro, in a controlled environment. Nowadays, advancements in technology allow us to create satisfactory eye models in terms of accuracy, which includes most of the anatomic structures of the human eye, and a lot of its functionality, such as accommodation, input light amount control, and visual processing. The use of rapid prototyping techniques such as additive manufacturing allows a relatively cheap eye model device to be ready for use in a very short time, and 3D modeling software is extremely useful for a quick design of a prototype, which can be later improved and adapted with little effort. Several off-the-shelf components are readily available, while more sophisticated and custom machined parts can also be made, but with some added costs.

However, eye models are still far away from being perfect. The possibility of “copying” the properties and features of the real eye to a model is much more complex than has been expected by many specialists. There are still many small-scale phenomena that impact visual quality and are not well understood, such as the dispersion variation inside the lens during accommodation, and thus are not yet addressed by eye models. 

A major limitation of eye models is that they do not account for inter-subject variability. While they are appropriate for evaluating conditions as they happen in the average population, it is still not possible to obtain an eye model that is accurate enough to study a specific subject in vitro and plan a possible customized treatment. Eye models perform quite well in testing IOLs, but soft CLs are still a challenge to evaluate due to their anatomic variance [37]. Thus, a subject-specific model would be highly desirable.

Another limitation of eye models for human vision is the light detection mechanisms at the retinal surface. The process of phototransduction is very complex, and optical effects in image quality such as the Stiles–-Crawford effect, photoreceptor density, and the curvature of the retina are extremely hard to reproduce in physical eye models through a CCD or CMOS sensor. The photodetector density can be properly modeled by using sensors with sufficiently low pixel size, but it is not possible yet to take into account the variations with eccentricity, other than by indirectly emulating them using image processing. The Stiles–Crawford effect dampens some of the aberrations of the eye and improves image quality off-axis. Ironically, a feature considered a limitation in [46] was actually the most accurately an eye model could simulate the Stiles–Crawford effect. Images captured by the artificial eye appeared to be darker near the outer area, which was caused by the incident angles on the curved fiber-optic bundle that refracted and emitted at larger angles with respect to the optical axis as the field-of-view increased. The effect was mitigated by using a tapered fiber bundle with all of the fibers oriented to be normal to the incident angles from any field-of-view. Nevertheless, the effect was still too apparent compared to the much weaker Stiles–Crawford effect. Another suggested method for modeling this effect is to use an apodised pupil [20]. As for the curvature of the retina, this problem is partially solved by using fiber-optics, or moving sensors along an arcuate path, but there is still room for improvement.

Other limitations of eye models are their inability to test different lacrimal conditions, pathologies such as fogging of the cornea or cataracts, corneal biomechanics, diseases related with intraocular pressure, or retinal degeneration. To study these conditions, much more complexity will be necessary in future eye models.

Finally, correct modeling of the visual processing requires a better understanding of image processing on the retina before transmission of the image to the brain. Unfortunately, the neural component of the visual system is far from being well-understood. One of the interesting problems concerning recording of the image on the retina is the role of the length of the photosensitive parts of photoreceptors, which is different from the thickness of the photosensitive part of a CCD or CMOS element. Another question is the fact that the photosensors are actually the innermost layer of the retina, which is occluded by the remaining layers and blood vessels. Whether these tridimensional aspects of the retina have any influence in the image quality, compared to a 2D sensing surface, has not been explained yet. However, the neural component of the visual process is beyond the scope of this review, which focused on the image-forming capabilities of the optomechanical models described. 

This review closes with some comments about the use of physical eye models for industrial applications. The context of industrial optical testing usually involves either artificial camera devices or human subjective inspection. To ensure reduced return rates and the related production and reputational costs, industry is investing in very sensitive inspection methods, which have ultimately pushed the standards to limits that might not have an impact on the user experience if they are not seen under the limits of resolution of the human eye. Artificial cameras can be rapid, and usually detect very fine details in the samples and might incur in the rejection of samples with unnoticeable defects to the human eye. However, highly sensitive tests using high-resolution cameras would detect defects that are not visible to the naked eye, and thus have no impact on the viewer’s quality, forcing the rejection of units that are apt for human use. On the other hand, subjective human inspection usually requires a longer time, is subject to a lower degree of consistency, and comprises tedious work that should, whenever possible, be replaced by automatic processes. Therefore, it is often desirable to have available a human resolution camera to help in reducing this number of unnecessary rejections in an objective manner, thus assisting or replacing human subjective inspectors. One way of achieving this is through an artificial eye system that is able to mimic and work under different conditions, including different corneal lens shapes and different internal mediums, thus reproducing well the contrast sensitivity function of the human eye and to provide images of objects, such as display units for human use, under conditions similar to the ones of a human eye using those devices. A wide-field customizable optomechanical model of the human eye would be able to challenge the inspection process within the ranges of resolution of the human eye, at different distances of inspection, and providing a physiological platform to contribute to the automatic inspection of parts in the industry within a physiological eye paradigm.

## 9. Conclusions

This review presented the structure, characteristics, purposes, and performance of several optomechanical eye models. Artificial eye models are devices used mainly for research applications, or tunning and calibration of optical correction or measuring devices. These have been used mainly for physiological applications, but also have potential applications in industry. While there are various different designs and degrees of complexity reported in the literature, a typical artificial eye models comprises of a cornea, pupil, crystalline lens, retina, and fluid filled internal cavities. Recent devices have provided more realistic ways of mimicking accommodation, simulating natural optical aberrations and the image formation at the retina, but there are still many challenges to overcome. Furthermore, most publications tend to have an optimistic view on their respective devices, which can complicate an honest comparison between them.

Image and video sensors are widely used in quality checkups, to help or replace a human inspector. Most modern equipment is high resolution, which is more than enough when the task is the same as performed by a human. Furthermore, if the object being inspected is to be “viewed” by a human, such as in a display, only defects that are detected by the human eye should be rejected. Therefore, using a high-resolution inspection device will reject more objects than needed, decreasing production output. On the other hand, artificial eye devices will inspect the object the same way a human observer would, thus rejecting only the defects that are perceptible by humans. Other possible applications include robotics or the analysis of human behavior during driving.

## Figures and Tables

**Table 1:** Publications involving physical eye models considered in this review. Patented inventions are at the end of the table. Publications considered relevant for human vision applications are highlighted in **bold**.

Publication (Year)	Reference
Gliddon (1929)	[3]
Arell (1978)	[4]
Heath (1987)	[5]
Rudnicka (1992)	[6]
Oshika (1996)	[7]
Pujol (1998)	[8]
Moreno-Barriuso (2000)	[9]
Trinavarat (2001)	[10]
Dubbelman (2001)	[11]
Barry (2001)	[12]
Pieh (2002)	[13]
Holladay (2002)	[14]
Cheng (2003)	[15]
Barbero (2003)	[16]
Letfullin (2005)	[17]
Rawer (2005)	[18]
Kawamorita (2005)	[19]
**Gobbi (2006)**	[20]
Galetskiĭ (2006)	[21]
De Castro (2007)	[22]
Norrbi (2007)	[23]
Fernández (2007)	[24]
Artigas (2007)	[25]
**Choi (2008)**	[26]
Terwee (2008)	[27]
Campbell (2008)	[28]
Eppig (2008)	[29]
**Barcik (2008)**	[30]
Maxwell (2009)	[31]
McKelvie (2009)	[32]
Pieh (2009)	[33]
Shen (2009)	[34]
Goncharov (2009)	[35]
Eppig (2009)	[36]
**Bakaraju (2010)**	[37]
Inoue (2011)	[38]
Birkner (2011)	[39]
**Ohnuma (2011)**	[40]
Kim (2011)	[41]
**Petelczyc (2011)**	[42]
Pepose (2012)	[43]
Montés-Micó (2012)	[44]
**Ackermann (2013)**	[45]
**Arianpour (2013)**	[46]
Gatinel (2013)	[47]
**Drauschke (2013)**	[48]
Ruiz-Alcocer (2014)	[49]
Carson (2014)	[50]
**Liang (2014)**	[51]
Xie (2014)	[52]
Fung (2015)	[53]
**Förster (2015)**	[54]
Santiago-Alvarado (2015)	[55]
Vega (2015)	[56]
Esteve-Taboada (2015)	[57]
Yusuf (2015)	[58]
Guerra (2015)	[59]
Domínguez-Vicent (2015)	[60]
Mao (2016)	[61]
**Petsch (2016)**	[62]
Winter (2016)	[63]
Coughlan (2017)	[64]
Son (2017)	[65]
**Alba-Bueno (2017)**	[66]
Al-Mohamedi (2018)	[67]
Petelczy (2019)	[68]
Gu (2020)	[69]
Regal (2020)	[70]
Chae (2020)	[71]
Wang (2021)	[72]
**Schneider (1996)** *	[73]
**Schneider (1997)** *	[74]
**Ohnuma (1999)** *	[75]
Sheehy (2002) *	[76]
Altmann (2003) *	[77]
Yamaguchi (2006) *	[78]
Niven (2006) *	[79]
**Ehrmann (2014)** *	[80]

* Inventions described in patent applications.

**Table 2:** Main applications of physical eye models, as reported in the publications reviewed.

Application	Literature References
**Evaluate intraocular lenses:**	[7,10,13,14,16,17,18,19,20,22,23,25,26,27,28,29,30,31,32,33,38,41,43,44,47,48,49,50,56,60,65,66,68,71,73,74,75,77,80]
**Evaluate spectacles/contact lenses:**	[5,17,37,38,42,74,75,76,77,80]
**Evaluate ophtalmic equipment:**	[6,15,17,24,34,35,53,59,64,67,78,79,80]
**Evaluate measurement methods:**	[9,11,12,22,39,52,58,59,61,72,80]
**Evaluate treatments:**	[45,70]
**Modeling optical conditions:**	[3,6,8,17,21,30,37,40,46,48,64,80]
**Simulate human visual conditions:**	[26,45,54,66]
**Modeling accomodation:**	[24,51,54,55,57,62,63]
**Modeling other eye behaviour**	[17,21,54,55,69]
**Research/optical design:**	[3,6,35,46,51,64]
**Teaching purposes:**	[4]

**Table 3:** Optical measurement modalities used on the devices included in this review, as reported by the respective publications. The purpose of such measurements could be either related to the main goal of the research study, or just for the optical characterization of the device, prior to its intended use.

Optical Principles Used	Literature References
**Wavefront measurements:**	[9,15,17,21,23,24,28,32,34,35,37,41,44,48,59,64]
**Double-pass measurements:** *	[6,8,9,35,42,57,59,72]
**Single-pass measurements:**	[3,5,7,9,13,14,16,18,19,20,25,26,27,28,29,30,31,33,37,40,41,42,43,45,46,47,48,49,50,51,54,55,56,60,62,65,66,68,69,70,71]
**Fundus imaging and retinoscopy**	[4,6,38,52,53,58,63]
**Scheimpflug/Purkinje imaging:**	[11,12,22]
**Optical biometry and OCT**	[39,52,61,67,72]
**Other:**	[10,52,70,76]

* Excluding wavefront measurements.

**Table 4:** Comparison between the single-pass models with image formation features discussed in this review.

Ref.	Strengths	Limitations
[37,80]	High resolution sensor Off-axis vision Curved retina Pressurized medium Multi-modal measurements Accurate cornea Cornea humidity/temperature control Tear modeling Phakic or aphakic configurations Position tracking Can test IOLs, CLs and spectacles	No dynamic components Author reported limitations: ○ Flat detector ○ Cone distribution not modeled ○ Not sufficiently subject-specific to test off-the-shelf lenses Requires upright operation Eye model is patented, may not be easy to replicate
[45]	High resolution sensor Cornea humidity/temperature control Phakic configuration	Same limitations as [37,80] Requires upright operation
[46]	Minimizes spherical aberration Off-axis vision Curved retina Phakic or aphakic with IOL	No dynamic components Requires discrete image correction Darkening around the edges Cornea is glued to holder No variable power (without disassembling)
[64]	Off-the-shelf components Smart fluid storage Off-axis vision Eye model well described	No dynamic components Unrealistic cornea shape Human vision not implemented yet Probably flat detector (when implemented)
[48]	IOL shift, tilt, decentration Alignment control Vibrational analysis Dispersion analysis Physical and physiological measurements Eye model well described	No dynamic components Bulky device Too much focused on IOL evaluations
[62]	3D printed enclosure Soft-matter-based Phakic configuration Tunable lens Tunable iris Off-axis vision Field curvature compensation	Unrealistic cornea shape (Cooke triplet) Anatomic components in wrong order
[54]	Tunable lens Simulates accommodation Simulates saccadic movements Potential applications in the industry	Too complex and specific Unrealistic in anatomic terms Unrealistic saccades (retina does not move) No variable power
[30]	IOL shift, tilt, decentration Simple, but configurable Off-axis vision (presumably) Curved retina MTF computation detailed	No dynamic components Unrealistic cornea shape (spherical) Requires relay optics Modified wet-cell-based setup
[20]	Simple, but configurable Compact and portable Methods used to cancel out the neural component	No dynamic components Requires relay optics Modified wet-cell-based setup
[23]	Compact and portable Based on the ISO standards Multi-modal measurements Publication is a reference in literature	No dynamic components Does not seem very configurable Few details reported about the model
[28]	Popular medium solution Multi-modal measurements	No dynamic componentsNot focused on single-pass measurementsRequires relay optics (single-pass)
[73,74]	IOL shift, tilt, decentration Retina can translate in two directions Off-axis vision Human observer with image capture in parallel Cornea can pressurized to avoid deformations Variable aperture iris (automatic) Adjustment by subject Can test IOLs, CLs and spectacles	No dynamic components Flat retina Details about optical components too vague Eye model is patented, may not be easy to replicate Publication is old
[77]	Curved retina Pressurized medium Multi-modal measurements Controlled environment Cornea humidity/temperature control Tear modeling Phakic or aphakic configurations Can test IOLs, CLs and spectacles	According to [80], incident angles at which wavefront measuring instruments can be used effectively are severely limited Requires relay optics (single-pass) Device assembly details too vague Eye model is patented, may not be easy to replicate Publication is old
[55]	Phakic configuration Light sensitive dynamic pupil Dynamic crystalline lens Mechanical analysis	Too much focused in finite element simulations Not clear if device was really implemented
[51]	Phakic configuration Dynamic crystalline lens Crystalline fabrication highly detailed	Too much focused on the crystalline lens Authors claim the system being too much aberrated
[40]	Corrects both spherical and chromatic aberrations Evaluates each cone wavelength separately	No dynamic components Requires relay optics Wet-cell-based setup
[42]	Phakic configuration Can test CLs and spectacles	No dynamic components Unrealistic cornea shape No internal fluid (used air) ○ Had to scale optical powers, distances and aperture diameter
[26]	Both spherical and chromatic aberrations modeled Polychromatic testing	No dynamic components Unrealistic cornea shape (two singlet lenses) Requires relay optics (presumably) Wet-cell-based setup
[27]	Cornea modeled from multiple subjects’ data Qualitative ray tracing analysis	No dynamic components Requires relay optics (presumably) Wet-cell-based setup
[75]	Embodiments’ dimensions are reported One of the embodiments has a phakic configuration Can test IOLs, CLs and spectacles Reports the IOL lens design procedure Tear modeling	No dynamic components Some of the embodiments are vaguely described One of the embodiments is a wet-cell-based setup Uses complex relay optics According to [80], depends on a wide-angle camera-type objective lens placed in front of the system to bring an image to focus over the flat area of the CCD camera sensor Eye model is patented, may not be easy to replicate Publication is old
[76]	Anterior segment independent of posterior segment Aqueous and vitreous fluids separated (different fluids can be used) Configurable anterior segment Off-axis vision Curved retina	No dynamic components Eye model 6.5 times the size of the natural eye Used only for radiation safety studies Sensor is away from retinal surface Eye model is patented, may not be easy to replicate
[36]	Pupil mimics accurately the “kappa” angle IOL shift, tilt, decentration	Wet-cell-based setup
[66]	Compares simulated objective measurements with subjective psychophysical measurements	Ordinary wet-cell model (see below)
[13]	Anterior and posterior chambers communicate through shunts Eye model description well detailed	Ordinary wet-cell model (see below)
Other *	Easy to replace components Validated by ISO standards Modifications can be applied ○ Cornea may be modified to match human eye characteristics Some models allow shift, decentration and tilt of IOLs	Too simplistic Usually require an optical bench No enclosure in most cases Not portable No dynamic components Usually requires relay optics Usually used only for IOL evaluation

* other wet-cell single-pass models.

## Data Availability

Not applicable.

## References

[1] Gullstrand A. (1909). Appendices II and IV. Helmholtz’s Handbuch der Physiologischen Optik.

[2] Peng J., Jia X., Wang J., Hu Y. (2019). A Simplifed Model Eye for Testing Fundus Imaging Device. Zhongguo Yi Liao Qi Xie Za Zhi.

[3] Gliddon G.H. (1929). An optical replica of the human eye for the study of the retinal image. Arch. Ophthalmol..

[4] Arell A., Kolari S. (1978). Experiments on a model eye. Am. J. Phys..

[5] Heath D.A., McCormack G.L., Vaughan W.H. (1987). Mapping of Ophthalmic Lens Distortions with a Pinhole Camera. Optom. Vis. Sci..

[6] Rudnicka A.R., Edgar D.F., Bennett A.G. (1992). Construction of a model eye and its applications. Ophthalmic Physiol. Opt..

[7] Oshika T., Shiokawa Y. (1996). Effect of folding on the optical quality of soft acrylic intraocular lenses. J. Cataract Refract. Surg..

[8] Pujol J., Arjona M., Arasa J., Badia V. (1998). Influence of amount and changes in axis of astigmatism on retinal image quality. J. Opt. Soc. Am. A.

[9] Moreno-Barriuso E., Navarro R. (2000). Laser Ray Tracing versus Hartmann–Shack sensor for measuring optical aberrations in the human eye. J. Opt. Soc. Am. A.

[10] Trinavarat A., Atchaneeyasakul L., Udompunturak S. (2001). Neodymium:YAG laser damage threshold of foldable intraocular lenses. J. Cataract Refract. Surg..

[11] Dubbelman M., Van der Heijde G.L. (2001). The shape of the aging human lens: Curvature, equivalent refractive index and the lens paradox. Vis. Res..

[12] Barry J.C., Dunne M., Kirschkamp T. (2001). Phakometric measurement of ocular surface radius of curvature and alignment: Evaluation of method with physical model eyes. Ophthalmic Physiol. Opt..

[13] Pieh S., Marvan P., Lackner B., Hanselmayer G., Schmidinger G., Leitgeb R., Sticker M., Hitzenberger C.K., Fercher A.F., Skorpik C. (2002). Quantitative performance of bifocal and multifocal intraocular lenses in a model eye: Point spread function in multifocal intraocular lenses. Arch. Ophthalmol..

[14] Holladay J.T., Piers P.A., Koranyi G., Van der Mooren M., Norrby N.E.S. (2002). A new intraocular lens design to reduce spherical aberration of pseudophakic eyes. J. Refract. Surg..

[15] Cheng X., Himebaugh N.L., Kollbaum P.S., Thibos L.N., Bradley A. (2003). Validation of a clinical Shack-Hartmann aberrometer. Optom. Vis. Sci..

[16] Barbero S., Marcos S., Jiménez-Alfaro I. (2003). Optical aberrations of intraocular lenses measured in vivo and in vitro. J. Opt. Soc. Am. A Opt. Image Sci. Vis..

[17] Letfullin R., Cherezova T., Belyakov A., Kudryashov A., Gruneisen M.T., Gonglewski J.D., Giles M.K. (2005). Human eye model based on bimorph flexible mirror. Optics and Photonics, Proceedings of the Advanced Wavefront Control: Methods, Devices, and Applications III, San Diego, CA, USA, 31 July–4 August 2005.

[18] Rawer R., Stork W., Spraul C.W., Lingenfelder C. (2005). Imaging quality of intraocular lenses. J. Cataract Refract. Surg..

[19] Kawamorita T., Uozato H. (2005). Modulation transfer function and pupil size in multifocal and monofocal intraocular lenses in vitro. J. Cataract Refract. Surg..

[20] Gobbi P.G., Fasce F., Bozza S., Brancato R. (2006). Optomechanical eye model with imaging capabilities for objective evaluation of intraocular lenses. J. Cataract Refract. Surg..

[21] Galetskiĭ S.O., Cherezova T.Y., Belyakov A.I., Kudryashov A.V. (2006). Creating a model of the human eye by the methods of adaptive optics. J. Opt. Technol..

[22] De Castro A., Rosales P., Marcos S. (2007). Tilt and decentration of intraocular lenses in vivo from Purkinje and Scheimpflug imaging. Validation study. J. Cataract Refract. Surg..

[23] Norrby S., Piers P., Campbell C., van der Mooren M. (2007). Model eyes for evaluation of intraocular lenses. Appl. Opt..

[24] Fernández E.J., Artal P. (2007). Dynamic eye model for adaptive optics testing. Appl. Opt..

[25] Artigas J.M., Menezo J.L., Peris C., Felipe A., Díaz-Llopis M. (2007). Image quality with multifocal intraocular lenses and the effect of pupil size: Comparison of refractive and hybrid refractive-diffractive designs. J. Cataract Refract. Surg..

[26] Choi J., Schwiegerling J. (2008). Optical performance measurement and night driving simulation of ReSTOR, ReZoom, and Tecnis multifocal intraocular lenses in a model eye. J. Refract. Surg..

[27] Terwee T., Weeber H., van der Mooren M., Piers P. (2008). Visualization of the retinal image in an eye model with spherical and aspheric, diffractive, and refractive multifocal intraocular lenses. J. Refract. Surg..

[28] Campbell C.E. (2008). Wavefront measurements of diffractive and refractive multifocal intraocular lenses in an artificial eye. J. Refract. Surg..

[29] Eppig T., Scholz K., Langenbucher A. (2008). Assessing the optical performance of multifocal (diffractive) intraocular lenses. Ophthalmic Physiol. Opt..

[30] Barcik A., Nowak J., Siedlecki D., Zając M., Zarówny J., Popiolek-Masajada A., Jankowska E., Urbanczyk W. (2008). Physical model of human eye with implantable intraocular lenses. Proceedings of the 16th Polish-Slovak-Czech Optical Conference on Wave and Quantum Aspects of Contemporary Optics.

[31] Maxwell W.A., Lane S.S., Zhou F. (2009). Performance of presbyopia-correcting intraocular lenses in distance optical bench tests. J. Cataract Refract. Surg..

[32] McKelvie J., Ku J.Y., McArdle B., McGhee C. (2009). Wavefront aberrometry: Comparing and profiling higher-order aberrations produced by intraocular lenses in vitro using a physical model eye system and Hartman-Shack aberrometry. J. Cataract Refract. Surg..

[33] Pieh S., Fiala W., Malz A., Stork W. (2009). In vitro strehl ratios with spherical, aberration-free, average, and customized spherical aberration-correcting intraocular lenses. Investig. Ophthalmol. Vis. Sci..

[34] Shen J., Thibos L.N. (2009). Measuring ocular aberrations and image quality in peripheral vision with a clinical wavefront aberrometer. Clin. Exp. Optom..

[35] Goncharov A.V., Lerat B., Nowakowski M., Dainty C., Bosse H., Bodermann B., Silver R.M. (2009). Inverse optical design: Building and testing an artificial eye. SPIE Europe Optical Metrology, Proceedings of the Modeling Aspects in Optical Metrology II, Munich, Germany, 14–18 June 2009.

[36] Eppig T., Scholz K., Löffler A., Meßner A., Langenbucher A. (2009). Effect of decentration and tilt on the image quality of aspheric intraocular lens designs in a model eye. J. Cataract Refract. Surg..

[37] Bakaraju R.C., Ehrmann K., Falk D., Ho A., Papas E. (2010). Physical human model eye and methods of its use to analyse optical performance of soft contact lenses. Opt. Express.

[38] Inoue M., Noda T., Mihashi T., Ohnuma K., Bissen-Miyajima H., Hirakata A. (2011). Quality of Image of Grating Target Placed in Model of Human Eye with Corneal Aberrations as Observed Through Multifocal Intraocular Lenses. Am. J. Ophthalmol..

[39] Birkner S., Einighammer J., Oltrup T., Bende T., Jean B. (2011). Biometric measurements inside the model eye using a two wavelengths Fourier domain low coherence interferometer. Biomed. Tech..

[40] Ohnuma K., Kayanuma H., Lawu T., Negishi K., Yamaguchi T., Noda T. (2011). Retinal image contrast obtained by a model eye with combined correction of chromatic and spherical aberrations. Biomed. Opt. Express.

[41] Kim M.J., Zheleznyak L., Macrae S., Tchah H., Yoon G. (2011). Objective evaluation of through-focus optical performance of presbyopia-correcting intraocular lenses using an optical bench system. J. Cataract Refract. Surg..

[42] Petelczyc K., Bará S., Lopez A.C., Jaroszewicz Z., Kakarenko K., Kolodziejczyk A., Sypek M. (2011). Imaging properties of the light sword optical element used as a contact lens in a presbyopic eye model. Opt. Express.

[43] Pepose J.S., Wang D., Altmann G.E. (2012). Comparison of Through-Focus Image Sharpness Across Five Presbyopia-Correcting Intraocular Lenses. Am. J. Ophthalmol..

[44] Montés-Micó R., López-Gil N., Pérez-Vives C., Bonaque S., Ferrer-Blasco T. (2012). In vitro optical performance of nonrotational symmetric and refractive-diffractive aspheric multifocal intraocular lenses: Impact of tilt and decentration. J. Cataract Refract. Surg..

[45] Ackermann R., Kammel R., Merker M., Kamm A., Tünnermann A., Nolte S. (2013). Optical side-effects of fs-laser treatment in refractive surgery investigated by means of a model eye. Biomed. Opt. Express.

[46] Arianpour A., Tremblay E.J., Stamenov I., Ford J.E., Schanzlin D.J., Lo Y. (2013). An optomechanical model eye for ophthalmological refractive studies. J. Refract. Surg..

[47] Gatinel D., Houbrechts Y. (2013). Comparison of bifocal and trifocal diffractive and refractive intraocular lenses using an optical bench. J. Cataract Refract. Surg..

[48] Drauschke A., Rank E., Traxler L., Lux K., Krutzler C. (2013). Mechanical Eye Model for Comparison of Optical and Physiological Imaging Properties. IFAC Proc. Vol..

[49] Ruiz-Alcocer J., Madrid-Costa D., García-Lázaro S., Ferrer-Blasco T., Montés-Micó R. (2014). Optical performance of two new trifocal intraocular lenses: Through-focus modulation transfer function and influence of pupil size. Clin. Exp. Ophthalmol..

[50] Hill W., Carson D., Hong X., Karakelle M. (2014). Optical bench performance of Acrysof^®^ IQ resTOr^®^, AT LISA^®^ tri, and FineVision^®^ intraocular lenses. Clin. Ophthalmol..

[51] Liang D., Xiang K., Du J.-W., Yang J.-N., Wang X.-Y. (2014). Biomimetic optical system using polymer lenses with tunable focus. Opt. Eng..

[52] Xie P., Hu Z., Zhang X., Li X., Gao Z., Yuan D., Liu Q. (2014). Application of 3-dimensional printing technology to construct an eye model for fundus viewing study. PLoS ONE.

[53] Fung T.H.M., Yusuf I.H., Xue K., Smith L.M., Patel C.K. (2015). Heidelberg Spectralis ultra-widefield fundus fluorescein angiography in infants. Am. J. Ophthalmol..

[54] Förster E., Stürmer M., Wallrabe U., Korvink J., Brunner R. (2015). Bio-inspired variable imaging system simplified to the essentials: Modelling accommodation and gaze movement. Opt. Express.

[55] Santiago-Alvarado A., Cruz-Félix A., Hernández Méndez A., Pérez-Maldonado Y., DomÍnguez-Osante C., George T., Dutta A.K., Islam M.S. (2015). Design and characterization of a tunable opto-mechatronic system to mimic the focusing and the regulation of illumination in the formation of images made by the human eye. SPIE Defense + Security, Proceedings of the Micro- and Nanotechnology Sensors, Systems, and Applications VII, Baltimore, MD, USA, 20–24 April 2015.

[56] Vega F., Alba-Bueno F., Millán M.S., Varón C., Gil M.A., Buil J.A. (2015). Halo and Through-Focus Performance of Four Diffractive Multifocal Intraocular Lenses. Investig. Ophthalmol. Vis. Sci..

[57] Esteve-Taboada J.J., Del Águila-Carrasco A.J., Marín-Franch I., Bernal-Molina P., Montés-Micó R., López-Gil N. (2015). Opto-mechanical artificial eye with accommodative ability. Opt. Express.

[58] Yusuf I.H., Fung T.H.M., Patel C.K. (2015). Ultra-widefield retinal imaging through a black intraocular lens. J. Cataract Refract. Surg..

[59] García-Guerra C.E., Aldaba M., Arjona M., Pujol J. (2015). Binocular open-view system to perform estimations of aberrations and scattering in the human eye. Appl. Opt..

[60] Domínguez-Vicent A., Esteve-Taboada J.J., Del Águila-Carrasco A.J., Ferrer-Blasco T., Montés-Micó R. (2016). In vitro optical quality comparison between the Mini WELL Ready progressive multifocal and the TECNIS Symfony. Graefes Arch. Clin. Exp. Ophthalmol..

[61] Mao X., Banta J.T., Ke B., Jiang H., He J., Liu C., Wang J. (2016). Wavefront Derived Refraction and Full Eye Biometry in Pseudophakic Eyes. PLoS ONE.

[62] Petsch S., Schuhladen S., Dreesen L., Zappe H. (2016). The engineered eyeball, a tunable imaging system using soft-matter micro-optics. Light Sci. Appl..

[63] Winter S., Sabesan R., Tiruveedhula P., Privitera C., Unsbo P., Lundström L., Roorda A. (2016). Transverse chromatic aberration across the visual field of the human eye. J. Vis..

[64] Coughlan M.F., Mihashi T., Goncharov A.V. (2017). Opto-mechanical design of a dispersive artificial eye. Appl. Opt..

[65] Son H.S., Tandogan T., Liebing S., Merz P., Choi C.Y., Khoramnia R., Auffarth G.U. (2017). In Vitro optical quality measurements of three intraocular lens models having identical platform. BMC Ophthalmol..

[66] Alba-Bueno F., Garzón N., Vega F., Poyales F., Millán M.S. (2018). Patient-Perceived and Laboratory-Measured Halos Associated with Diffractive Bifocal and Trifocal Intraocular Lenses. Curr. Eye Res..

[67] Al-Mohamedi H., Kelly-Pérez I., Prinz A., Oltrup T., Leitritz M., Cayless A., Bende T. (2019). A systematic comparison and evaluation of three different Swept-Source interferometers for eye lengths biometry. Z. Med. Phys..

[68] Petelczyc K., Kolodziejczyk A., Błocki N., Byszewska A., Jaroszewicz Z., Kakarenko K., Kołacz K., Miler M., Mira-Agudelo A., Torres-Sepúlveda W. (2020). Model of the light sword intraocular lens: In-Vitro comparative studies. Biomed. Opt. Express.

[69] Gu L., Poddar S., Lin Y., Long Z., Zhang D., Zhang Q., Shu L., Qiu X., Kam M., Javey A. (2020). A biomimetic eye with a hemispherical perovskite nanowire array retina. Nature.

[70] Regal S., Troughton J., Delattre R., Djenizian T., Ramuz M. (2020). Changes in temperature inside an optomechanical model of the human eye during emulated transscleral cyclophotocoagulation. Biomed. Opt. Express.

[71] Chae S.H., Son H.S., Khoramnia R., Lee K.H., Choi C.Y. (2020). Laboratory evaluation of the optical properties of two extended-depth-of-focus intraocular lenses. BMC Ophthalmol..

[72] Wang H., Liu W., Hu Z., Li X., Li F., Duan L. (2021). Model eye tool for retinal optical coherence tomography instrument calibration. J. Innov. Opt. Health Sci..

[73] Schneider R.T., Keates R.H. (1996). Apparatus and Methods for Evaluating Vision through an Intraocular Lens. U.S. Patent.

[74] Schneider R.T., Keates R.H. (1997). Vision Simulating Apparatus and Method. U.S. Patent.

[75] Ohnuma K., Qi H., Minato A. (1999). Ocular Optical System Simulation ApparatuS. U.S. Patent.

[76] Sheehy J.B., Gish K.W., Sprenger J.J. (2002). Artificial Human Eye and Test Apparatus. U.S. Patent.

[77] Altmann G.E. (2003). Lens-Eye Model and Method for Prfdicting In-Vivo Lens Performance. U.S. Patent.

[78] Yamaguchi T., Nakazaw N., Toshifumi M., Hirohara Y. (2006). Model Eye for Eye Characteristic Measuring Device and Calibration Method for The Same. U.S. Patent.

[79] Niven G.D. (2006). Eye Model for Measurement. U.S. Patent.

[80] Ehrmann K., Bakaraju R.C., Falk D. (2014). Physical Model Eye Systems and Methods. U.S. Patent.

[81] Bennett A.G., Rabbetts R.B. (1989). Clinical Visual Optics.

[82] Portney V. (1992). Optical testing and inspection methodology for modern intraocular lenses. J. Cataract Refract. Surg..

[83] (1999). Ophthalmic Implants—Intraocular Lenses—Part 2: Optical Properties and Test Methods.

[84] Bajrovic S., Zimmermann D. (2018). ISO-compliant IOL inspection Implementing the ISO-compliant Intraocular Lens (IOL) inspection in your production. GlobalCONTACT.

[85] (2014). Ophthalmic Implants—Intraocular Lenses—Part 2: Optical Properties and Test Methods.

[86] Swift A., Gooi P. (2020). Artificial eye models: An opportunity to increase surgical training exposure in ophthalmology during and beyond the COVID-19 pandemic. J. Clin. Ophthalmol..

[87] Swift A., Waldner D., Gorner A., Chung H., Ahmed Y., Docherty G., Gooi P. (2021). Face and content validity of an artificial eye model for Ab-Interno Goniotomy. Eur. J. Ophthalmol..

[88] Wang D., Mulvey F.B., Pelz J.B., Holmqvist K. (2017). A study of artificial eyes for the measurement of precision in eye-trackers. Behav. Res. Methods.

[89] Gu J.J., Meng M., Cook A., Liu P.X. (2006). Design, sensing and control of a robotic prosthetic eye for natural eye movement. Appl. Bionics Biomech..

[90] Model Eye for Ultrasonic Biometry. 3B Scientific. Item No. 1012869 [U10018]. https://www.3bscientific.com/us/model-eye-for-ultrasonic-biometry-1012869-u10018-3b-scientific,p_838_18625.html.

[91] Díaz-Doutón F., Benito A., Pujol J., Arjona M., Güell J.L., Artal P. (2006). Comparison of the retinal image quality with a Hartmann-Shack wavefront sensor and a double-pass instrument. Investig. Ophthalmol. Vis. Sci..

[92] Escudero-Sanz I., Navarro R. (1999). Off-axis aberrations of a wide-angle schematic eye model. J. Opt. Soc. Am. A Opt. Image Sci. Vis..

[93] Liou H.L., Brennan N.A. (1997). Anatomically accurate, finite model eye for optical modeling. J. Opt. Soc. Am. A Opt. Image Sci. Vis..

[94] Le Grand Y., Hage S.G. (1980). El Physiological Optics.

[95] Kirschkamp T., Jöckel M., Wählisch G., Barry J.C. (1998). Konstruktion eines Modellauges zur Simulation von Purkinje-Spiegelbildern für die Bestimmung der Krümmungsradien und der Lage der Augenlinse-Construction of a Model Eye to Simulate Purkinje Reflections for the Determination of the Radii of Curvature and of the Position of the Crystalline Lens of the Eye. Biomed. Tech. Eng..

[96] Navarro R., Santamaría J., Bescós J. (1985). Accommodation-dependent model of the human eye with aspherics. J. Opt. Soc. Am. A.

[97] Gobbi P.G., Fasce F., Bozza S., Brancato R., Manns F., Söderberg P.G., Ho A. (2003). Experimental characterization of the imaging properties of multifocal intraocular lenses. Biomedical Optics, Proceedings of the Ophthalmic Technologies XIII, San Jose, CA, USA, 25–31 January 2003.

[98] Panos J.G., Ho A., Ehrmann K., Bakaraju R.C. (2019). An Optically Equivalent Physical Eye Model for In-Vitro Assessment of Intraocular Lenses. Investig. Ophthalmol. Vis. Sci..

[99] Arianpour A., Tremblay E., Ford J., Lo Y. (2011). Optomechanical Fluid-filled Model of the Human Eye. Proceedings of the Optics in the Life Sciences.

[100] Xiong Y., Li J., Wang N., Liu X., Wang Z., Tsai F.F., Wan X. (2017). The analysis of corneal asphericity (Q value) and its related factors of 1,683 Chinese eyes older than 30 years. PLoS ONE.

[101] Dubbelman M., Sicam V.A.D.P., Van der Heijde G.L. (2006). The shape of the anterior and posterior surface of the aging human cornea. Vis. Res..

[102] Dubbelman M., Weeber H.A., van der Heijde R.G.L., Völker-Dieben H.J. (2002). Radius and asphericity of the posterior corneal surface determined by corrected Scheimpflug photography. Acta Ophthalmol. Scand..

[103] Haigis W., Lege B., Miller N., Schneider B. (2000). Comparison of immersion ultrasound biometry and partial coherence interferometry for intraocular lens calculation according to Haigis. Graefes Arch. Clin. Exp. Ophthalmol..

[104] Atchison D.A., Markwell E.L., Kasthurirangan S., Pope J.M., Smith G., Swann P.G. (2008). Age-related changes in optical and biometric characteristics of emmetropic eyes. J. Vis..

[105] Richdale K., Bullimore M.A., Zadnik K. (2008). Lens thickness with age and accommodation by optical coherence tomography. Ophthalmic Physiol. Opt..

[106] Praveen M.R., Vasavada A.R., Shah S.K., Shah C.B., Patel U.P., Dixit N.V., Rawal S. (2009). Lens thickness of Indian eyes: Impact of isolated lens opacity, age, axial length, and influence on anterior chamber depth. Eye.

[107] Manns F., Fernandez V., Zipper S., Sandadi S., Hamaoui M., Ho A., Parel J.M. (2004). Radius of curvature and asphericity of the anterior and posterior surface of human cadaver crystalline lenses. Exp. Eye Res..

[108] Ji S., Ponting M., Lepkowicz R.S., Rosenberg A., Flynn R., Beadie G., Baer E. (2012). A bio-inspired polymeric gradient refractive index (GRIN) human eye lens. Opt. Express.

[109] Martinez E.J.F., Soriano P.A. (2018). Variable-Power Accommodative Intraocular Lens and Assembly of Variablepower Accommodative Intraocuilar Lens and Capsular Ring. U.S. Patent.

[110] Carpi F., Frediani G., Turco S., De Rossi D. (2011). Bioinspired Tunable Lens with Muscle-Like Electroactive Elastomers. Adv. Funct. Mater..

[111] Leung C.K., Palmiero P.-M., Weinreb R.N., Li H., Sbeity Z., Dorairaj S., Leung D., Liu S., Liebmann J.M., Congdon N. (2010). Comparisons of anterior segment biometry between Chinese and Caucasians using anterior segment optical coherence tomography. Br. J. Ophthalmol..

[112] Li Q., Zong Y., Wen H., Yu J., Zhou C., Jiang C., Liu G., Sun X. (2021). Measurement of Iris Thickness at Different Regions in Healthy Chinese Adults. J. Ophthalmol..

[113] Schuhladen S., Preller F., Rix R., Petsch S., Zentel R., Zappe H. (2014). Iris-like tunable aperture employing liquid-crystal elastomers. Adv. Mater..

[114] Kimmle C., Schmittat U., Doering C., Fouckhardt H. (2011). Compact dynamic microfluidic iris for active optics. Microelectron. Eng..

[115] Wässle H., Boycott B.B. (1991). Functional architecture of the mammalian retina. Physiol. Rev..

[116] Polyak S.L. (1941). The Retina: The Anatomy and the Histology of the Retina in Man, Ape, and Monkey, Including the Consideration of Visual Functions, the History of Physiological Optics, and the Histological Laboratory Technique.

[117] Guenter B., Joshi N., Stoakley R., Keefe A., Geary K., Freeman R., Hundley J., Patterson P., Hammon D., Herrera G. (2017). Highly curved image sensors: A practical approach for improved optical performance. Opt. Express.

[118] Jung I., Xiao J., Malyarchuk V., Lu C., Li M., Liu Z., Yoon J., Huang Y., Rogers J.A. (2011). Dynamically tunable hemispherical electronic eye camera system with adjustable zoom capability. Proc. Natl. Acad. Sci. USA.

[119] Lee K.E., Klein B.E.K., Klein R., Quandt Z., Wong T.Y. (2009). Association of age, stature, and education with ocular dimensions in an older white population. Arch. Ophthalmol..

[120] Liu X., Zhang Y., Li L., Bian A., Zhou Q. (2019). Iris lens distance to predict the risk of intraocular pressure elevation after dark room provocative test. Graefes Arch. Clin. Exp. Ophthalmol..

[121] Nover A., Grote W. (1965). On the determination of the length of the axis of the human eye with ultrasound in the living person. Albrecht Von Graefes Arch. Klin. Exp. Ophthalmol..

[122] Rieger C.J., Carpenter F.G. (1959). Light scattering by commercial sugar solutions. J. Res. Natl. Bur. Stand. Sect. A Phys. Chem..

[123] Wagholikar N.K., Jagtap S.B. (2017). Optical Characterization of Sucrose (C_12_H_21_O_11_) at Different Temperatures. IJIRSET.

[124] Thibos L.N., Ye M., Zhang X., Bradley A. (1992). The chromatic eye: A new reduced-eye model of ocular chromatic aberration in humans. Appl. Opt..

[125] Schwiegerling J. (2004). Field Guide to Visual and Ophthalmic Optics.

[126] Wang K., Venetsanos D.T., Hoshino M., Uesugi K., Yagi N., Pierscionek B.K. (2020). A Modeling Approach for Investigating Opto-Mechanical Relationships in the Human Eye Lens. IEEE Trans. Biomed. Eng..

[127] Atchison D.A., Thibos L.N. (2016). Optical models of the human eye. Clin. Exp. Optom..

[128] Lang A.J., Lakshminarayanan V., Portney V. (1993). Phenomenological model for interpreting the clinical significance of the in vitro optical transfer function. J. Opt. Soc. Am. A.

[129] Piers P.A., Norrby N.E.S., Mester U. (2004). Eye models for the prediction of contrast vision in patients with new intraocular lens designs. Opt. Lett..

[130] Li G., Zwick H., Stuck B., Lund D.J. (2000). On the use of schematic eye models to estimate retinal image quality. J. Biomed. Opt..

[131] Dalimier E., Dainty C. (2008). Use of a customized vision model to analyze the effects of higher-order ocular aberrations and neural filtering on contrast threshold performance. J. Opt. Soc. Am. A.

